# Schisandrin B Attenuates Diabetic Cardiomyopathy by Targeting MyD88 and Inhibiting MyD88‐Dependent Inflammation

**DOI:** 10.1002/advs.202202590

**Published:** 2022-09-30

**Authors:** Wu Luo, Ke Lin, Junyi Hua, Jibo Han, Qiuyan Zhang, Lingfeng Chen, Zia A. Khan, Gaojun Wu, Yi Wang, Guang Liang

**Affiliations:** ^1^ Chemical Biology Research Center School of Pharmaceutical Sciences Wenzhou Medical University Wenzhou Zhejiang 325035 China; ^2^ School of Pharmaceutical Sciences Hangzhou Medical College Hangzhou Zhejiang 311399 China; ^3^ Department of Cardiology and Medical Research Center the First Affiliated Hospital Wenzhou Medical University Wenzhou Zhejiang 325035 China; ^4^ Department of Cardiovascular Medicine the Second Affiliated Hospital of Zhejiang University of Traditional Chinese Medicine Hangzhou Zhejiang 310009 China; ^5^ Department of Cardiovascular Medicine Quzhou Hospital of Traditional Chinese Medicine (Four Provincial Marginal Hospitals of Traditional Chinese Medicine Affiliated to Zhejiang University of Traditional Chinese Medicine) Quzhou Zhejiang 324002 China; ^6^ Department of Pathology and Laboratory Medicine University of Western Ontario London Ontario N6A 5C1 Canada

**Keywords:** cardiomyocytes, diabetic cardiomyopathy, inflammation, MyD88, Schisandrin B

## Abstract

Diabetes manifests as chronic inflammation and leads to the development diabetic cardiomyopathy (DCM). Targeting key proteins in inflammatory signaling may provide new therapy for DCM. In this study, the authors explore the pharmacological effects and mechanisms of Schisandrin B (Sch B), a natural compound with anti‐inflammatory activity against DCM. It is shown that Sch B prevents high‐level glucose (HG)‐induced hypertrophic and fibrotic responses in cultured cardiomyocytes. RNA sequencing and inflammatory qPCR microarray show that Sch B mainly affects myeloid differentiation primary response 88 (MyD88)‐dependent inflammatory gene expression in HG‐challenged cardiomyocytes. Further studies indicate that Sch B directly binds to and inhibits MyD88 activation, but does not alter MyD88‐independent Toll‐like receptor signaling in vivo and in vitro. Inhibiting or silencing MyD88 is associated with reduced levels of HG‐induced inflammatory cytokines and myocardial injuries in vitro. Treatment of type 1 and type 2 diabetic mice with Sch B protects heart function, reduces myocardial injuries, and decreases secretion of inflammatory cytokines. Cardiomyocyte‐specific MyD88 knockout also protects mice against cardiac inflammation and injury in type 1 diabetic mice. In conclusion, these studies show that cardiomyocyte MyD88 plays an apathogenetic role in DCM and Sch B specifically targets MyD88 to reduce inflammatory DCM.

## Graphical abstract

Hyperglycemia/high‐concentration glucose (HG) activates Toll‐like receptors (TLRs)‐MyD88 in cardiomyocytes and heart tissues. MyD88‐mediated activation of TAK1‐MAPKs/NF‐*κ*B signaling causes increased production of inflammatory factors, leading to cardiomyocyte hypertrophy and fibrosis. Schisandrin B is able to bind to the TIR domain of MyD88, potentially through Thr‐272 and Arg‐288 sites. Such binding suppresses TAK1‐MAPKs/NF‐*κ*B activation and attenuates inflammatory diabetic cardiomyopathy.

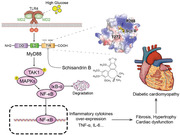

## 1 Introduction

Diabetic cardiomyopathy (DCM) is a prevalent diabetes‐induced secondary complication that leads to heart failure.^[^ ^1^ ^]^ This unique disease entity is presented as left ventricular dysfunction, independent of atherosclerosis, coronary heart disease, or hypertension.^[^ ^2^ ^]^ Cardiac dysfunction is often silent in diabetic patients and not detected until later stages. Unfortunately, it is estimated that close to half of asymptomatic/normotensive patients with well‐controlled diabetes have cardiac dysfunction.^[^ ^3^ ^]^ Effective treatment options are scarce. Regardless of whether morphological defects precede functional deficits or vice versa, understanding the pathophysiological mechanisms that lead to diabetic heart disease is critical to developing effective treatments.

Chronic inflammation in diabetes leads or contributes to DCM. Many inflammatory cytokines, such as tumor necrosis factor‐*α* (TNF*α*), interleukin‐6 (IL‐6), and interferon‐*γ* (IFN*γ*), have been reported to be elevated in diabetic models^[^ ^4^ ^]^ and can perpetuate excessive tissue remodeling,^[^ ^5^ ^]^ and in turn, the development of cardiac fibrosis leading to stiffness.^[^ ^6^ ^]^ These cytokines also play a rather important role through a positive feedback loop, primarily involving the nuclear factor‐*κ*B (NF‐*κ*B) activation.^[^ ^7^ ^]^ There certainly is promise in anti‐inflammatory therapy for diabetic patients to reduce cardiovascular complications, though further studies and better targets are needed. Toll‐like receptor 4 (TLR4), a component of the innate immune system, is thought to contribute to DCM by initiating hyperglycemia‐induced proinflammatory cascades. Diabetic mice and rats also show increased TLR4 levels in heart tissues. Our previous study demonstrated that advanced glycation end products (AGEs) directly bind TLR4 and its co‐receptor, myeloid differentiation factor 2 (MD2). Upon AGEs binding to MD2‐TLR4 in cardiomyocytes, the complex recruits two major adaptor molecules, myeloid differentiation primary response‐88 (MyD88) and TIR‐domain‐containing adapter‐inducing interferon‐*β* (TRIF), leading to the activation of downstream signaling pathways for the production of proinflammatory cytokines. Therefore, inhibition of key proteins in this signaling cascade may confer significant protection against inflammatory DCM.

**Figure 1:**
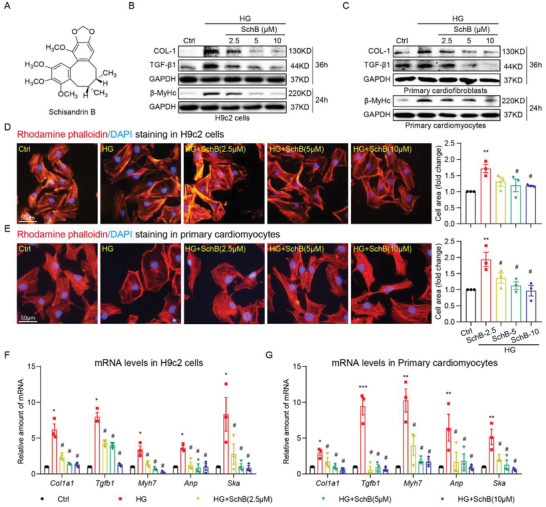
Sch B reduces HG‐induced cardiomyocyte hypertrophy and fibrotic responses. A) The chemical structure of Schisandrin B. B) Immunoblot analysis for *β*‐MyHc, Col‐1, and TGF‐*β*1 in H9C2 cells exposed to 33 × 10^−3^ m glucose (HG). Cells were pretreated with Sch B at indicated concentrations for 1 h and then stimulated with HG for 24 or 36 h. GAPDH was used as loading control. C) Immunoblot analysis for fibrosis markers in primary cardiac fibroblasts and hypertrophy marker in primary cardiomyocytes. Cells were pretreated with Sch B for 1 h before exposure to HG for 24 or 36 h. GAPHD was used as control. D,E) Rhodamine phalloidin staining of D) H9C2 and E) primary cardiomyocytes, showing the effect of Sch B on HG‐induced hypertrophy. Cells were treated as indicated for panel (B). Cells were counterstained with DAPI. Right panel shows the quantification of cell size, respectively [scale bar = 50 µm]. F,G) qPCR analysis of hypertrophic/fibrotic response genes in F) H9C2 cells and G) primary cardiomyocytes. Cells were pretreated with Sch B for 1 h and then stimulated with HG for 12 h. Levels of *Col1a1, Tgfb1, Myh6, Anp*, and *Ska* were normalized to *Actb*. Data in panels (D)–(G) shown as mean ± SEM (*n* = 3; *p < 0.05, **p < 0.01, ***p < 0.001, compared to Ctrl and #*p* < 0.05 compared to HG by one‐way ANOVA followed by Bonferroni's multiple comparisons test).

Schisandrin B (Sch B, **Figure** **1**A) is one of the most abundant bioactive dibenzocyclooctadiene derivatives found in the fruit of *Schisandra chinensis*.^[^ ^8^ ^]^ Studies have shown that Sch B mediates multiple biological activities in different disease models.^[^ ^9^ ^]^ In term of cardiac actions, Sch B has been shown to protect against ischemia‐reperfusion injury^[^ ^10^ ^]^ and doxorubicin‐induced cardiotoxicity,^[^ ^11^ ^]^ by reducing oxidative stress. In addition, Sch B can effectively prevent inflammatory responses through multiple mechanisms, including the suppression of proinflammatory cytokines TNF‐*α* and IL‐6.^[^ ^12^ ^]^ Despite these promising results, the underlying mechanisms and direct targets of Sch B have not been identified. Furthermore, the activity of Sch B has not been tested in DCM.

Based on its effects on other types of cardiac injury, in the present study, we investigated whether Sch B exhibits cardioprotective property in diabetes. We explored the underlying mechanisms and molecular targets of Sch B. We showed that Sch B suppresses high‐concentration glucose (HG)‐induced inflammation, hypertrophy, and fibrosis in cardiomyocytes, which are mediated by the ability of Sch B to directly bind and inhibit MyD88, an essential adaptor protein in TLR4 signaling pathway. Cardiomyocyte‐specific MyD88‐knockout mice were used to validate the importance of MyD88 in DCM. We also found that treatment of type 1 and 2 diabetic mice with Sch B led to reduced cardiac dysfunction. Importantly, our studies have uncovered MyD88 as a novel mechanism of DCM and a specific target of Sch B.

## 2 Results and Discussion

### 2.1 Sch B Prevents HG‐Induced Hypertrophic and Fibrotic Responses in Cardiac Cells

We first examined whether Sch B is able to suppress the well‐documented effects of high levels of glucose on cardiac cells. We first showed that rat cardiomyocyte‐like H9C2 cells readily take up exogenous Sch B from the culture media (Figure S1A,B, Supporting Information). No significant toxicity was observed in the cells upon treatment with 10 × 10^−6^ m Sch B (Figure S1C, Supporting Information). We then treated H9C2 cells with Sch B at 2.5 × 10^−6^, 5 × 10^−6^, and 10 × 10^−6^ m for 1 h before exposing the cells to HG (33 × 10^−3^ m glucose). Immunoblotting for surrogate markers of hypertrophy and fibrosis revealed that Sch B dose‐dependently prevents HG‐induced cardiac *β*‐MyHc (Figure 1B), as well as collagen 1 (Col‐1) and TGF‐*β*1 (Figure 1B). We also determined whether primary cardiac cells also show protection from HG when treated with Sch B. Our results showed that Sch B prevents HG‐induced *β*‐MyHc in rat primary cardiomyocytes and fibrotic proteins Col‐1 and TGF‐*β*1 in cardiac fibroblasts (Figure 1C). Next, we confirmed cardiomyocyte hypertrophy by staining both H9C2 cells and primary cardiomyocytes with rhodamine phalloidin to measure cell size. As expected, based on our immunoblotting, we observed increased cell size with the exposure of HG (Figure 1D,E), while this increase was reversed in cells pretreated with Sch B. Consistent with these data, mRNA levels of hypertrophic and fibrotic genes were induced by HG but not upon Sch B treatment (Figure 1F,G). These data showed that Sch B protects cardiac cells against HG‐induced hypertrophy and excessive matrix protein production.

### 2.2 Sch B Inhibits MyD88‐Dependent Inflammation in HG‐Challenged Cardiac Cells

**Figure 2:**
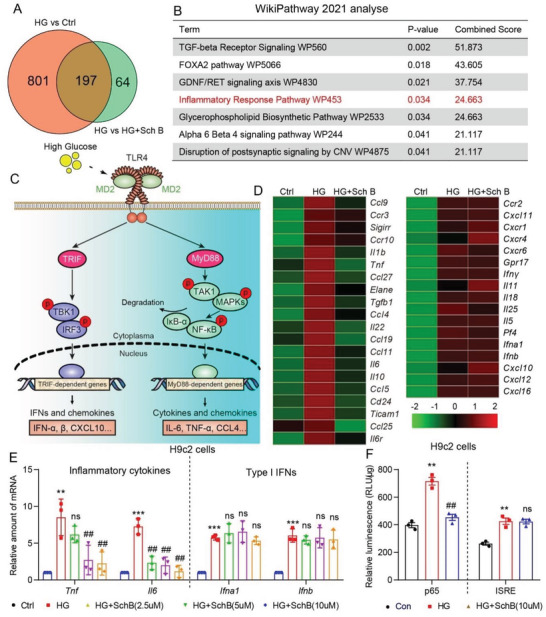
Sch B regulates the expression of inflammatory cytokines in H9C2 cells. A,B) H9C2 cells were pretreated with 10 × 10^−6^ m Sch B for 1 h before exposure to HG for 6 h. RNA sequencing was carried out and differentially expressed genes were identified by DESeq 2. Panel (A) shows the Venn diagram of differentially expressed genes between control versus HG and HG versus HG+Sch B. WikiPathway enrich analysis of overlap of differentially expressed genes in plane B. The inflammatory response pathway is highlighted by red. C) Schematic illustrating HG‐induced innate toll‐like receptor‐4 (TLR4) signaling leading to the induction of inflammatory factors. D) Heat‐map of inflammation cytokines detected by qPCR assay. H9C2 cells were pretreated with 10 × 10^−6^ m Sch B for 1 h and then exposed to HG for 8 h. Left panel shows inflammation cytokines that were suppressed by Sch B pretreatment. Right panel shows factors that were not suppressed by Sch B. E) qPCR verification of classical TLR4 proinflammatory cytokines (TNF‐*α* and IL6) and type 1 interferons (IFN‐*α* and ‐*β*) in H9C2 cells pretreated with Sch B before HG exposure. Cells were treated as indicated for panel (D). F) NF‐*κ*B and IRES reporter activity in H9C2 cells exposed to HG, with or without Sch B pretreatment. Data in panels (E) and (F) are shown as mean ± SEM (*n* = 3; **p < 0.01, ***p < 0.001 compared to Ctrl and ##*p* < 0.01, ns = not significant, compared to by one‐way ANOVA followed by Bonferroni's multiple comparisons test).

To build on our findings and to identify how Sch B may be protecting cardiac cells, we performed RNA‐sequencing to highlight differentially expressed genes. We found that 197 genes are potentially regulated by Sch B in HG‐challenged cardiomyocytes (**Figure** **2**A and Figure S2A–C, Supporting Information). Interestingly, pathway enrichment analysis showed that the inflammatory response is mainly involved in the activity of Sch B in cardiomyocytes (Figure 2B). To determine whether Sch B regulates HG‐induced inflammatory response, we further performed a quantitative polymerase chain reaction (qPCR) array for 120 inflammatory genes on RNA isolated from H9C2 cells challenged with HG, with or without Sch B pretreatment. We have previously shown that HG activates TLR4‐mediated inflammation in cardiomyocytes to induce inflammatory factor production^[^ ^4b^ ^]^ (Figure 2C). TLR4 mediates inflammatory signals through a MyD88‐dependent and/or a TRIF‐dependent pathway in innate immunity.^[^ ^13^ ^]^ Surprisingly, our results showed that Sch B preferentially downregulates MyD88‐dependent cytokines, and not TRIF‐dependent type 1 interferons (Figure 2D). Real‐time qPCR assay for MyD88‐dependent *Tnfa* and *Il6* and TRIF‐dependent *Ifna1* and *Ifnb* confirmed this phenomenon (Figure 2E). Similar results were also obtained in rat primary cardiomyocytes (Figure S3A, Supporting Information). We confirmed the inhibitory activity of Sch B on TNF‐*α* and IL‐6 by measuring the cytokines in culture media of H9C2 cells (Figure S3B, Supporting Information). Next, we confirmed how Sch B differentially regulates MyD88‐ and TRIF‐mediated transcriptional factors by probing for NF‐*κ*B (MyD88 pathway) and interferon‐stimulated response element (ISRE; TRIF pathway) activity, respectively. H9C2 cells were transfected with GFP reporters and exposed to HG, with or without Sch B pretreatment. We showed that Sch B suppresses HG‐induced NF‐*κ*B but not the TRIF‐dependent interferon pathway (Figure 2F).

### 2.3 Sch B Inhibits MyD88‐Dependent Signaling Pathway in HG‐Challenged Cardiac Cells

**Figure 3:**
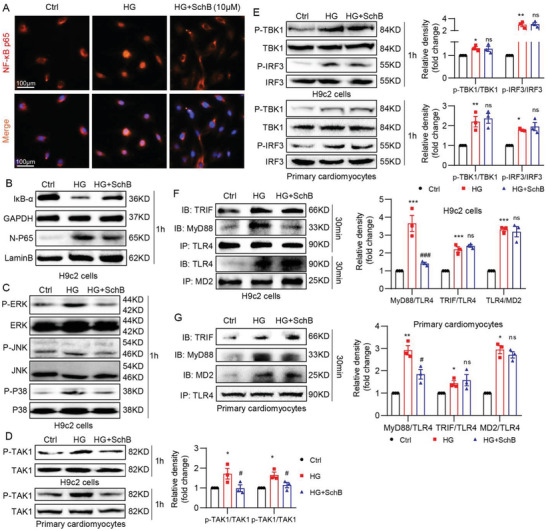
Sch B exhibits differential regulation of TLR4/TRIF and TLR4/MyD88 pathways. A) Immunofluorescence staining of H9C2 cells for p65 subunit of NF‐*κ*B (red). Cells were pretreated with 10 × 10^−6^ m Sch B for 1 h and then exposed to HG for 1 h. Cells were counterstained with DAPI (blue) (scale bar = 100 µm). B) Immunoblot analysis of I*κ*B*α* in total cell lysates and p65 in nuclear fractions prepared from H9C2 cells. Cells were treated as indicated for panel (A). GAPDH was used as control for total cell lysates and lamin B for nuclear proteins. C) Western blot analysis of MAPK activation in H9C2 cells. Cells were treated as in panel (B). Total cell lysates were probed for phosphorylated (p‐) and total ERK, JNK, and p38. D) Levels of MyD88 pathway protein TAK1 in H9C2 cells exposed to HG. Cells were treated as indicated for panel (B). Upper blots showing H9C2 cells and lower blots showing primary cardiomyocytes. Densitometric quantification of blots is shown in right panel. E) Analysis of TRIF pathway showing levels of TBK1 and IRF3. H9C2 (upper blots) and primary cardiomyocytes (lower blots) were treated as indicated for panel (B). Densitometric quantification is shown on right. F) Co‐immunoprecipitation analysis to examine the MD2/TLR4, TLR4/TRIF, and TLR4/MyD88 complexes in H9C2 cells. Cells were pretreated with 10 × 10^−6^ m Sch B for 1 h and then exposed to HG for 30 min. TLR4 was immunoprecipitated (IP) and TRIF and MyD88 were detected by immunoblotting (IB). In lower blots, MD2 was immunoprecipitated (IP) and TLR4 was detected by immunoblotting (IB). G) Co‐immunoprecipitation of MD2/TLR4, TLR4/TRIF, and TLR4/MyD88 complexes in primary cardiomyocytes. Cells were treated as indicated for panel (F). Densitometric quantification is shown in the lower panel. All data in panels (D)–(G) is shown as mean ± SEM (*n* = 3; *p < 0.05, **p < 0.01 compared to Ctrl and ##*p* < 0.01, ns = not significant compared to HG by one‐way ANOVA followed by Bonferroni's multiple comparisons test).

HG induces both MyD88‐ and TRIF‐dependent pathways in cardiac cells (Figure 2C), and our data illustrated that the protection mediated by Sch B may be primarily through suppression of the MyD88 pathway. We further validated the selectivity of Sch B for cardiac cells by probing the signaling proteins in both MyD88‐ and TRIF‐dependent pathways. Staining assay for p65 subunit of NF‐*κ*B (**Figure** **3**A) and immunoblotting assay for inhibitor of *κ*B (I*κ*B‐*α*) and nuclear p65 (Figure 3B and Figure S4A, Supporting Information) confirmed that Sch B pretreatment suppressed HG‐induced NF‐*κ*B activation in H9C2 cells. Other signaling proteins in the MyD88 pathway, such as MAPKs and TAK1, followed the same pattern: activation by HG and suppression by Sch B pretreatment (Figure 3C,D and Figure S4B, Supporting Information). As would be expected from the ISRE reporter study above, Sch B failed to suppress the TRIF‐pathway, as can be seen by immunoblotting of p‐TBK1 and p‐IRF3 in both H9C2 cells and primary cardiomyocytes (Figure 3E). To strengthen our conclusion that Sch B targets the MyD88 pathway in HG‐challenged cardiac cells, we immunoprecipitated MD2 and TLR4 to determine the interaction between MyD88 and TRIF proteins. We showed that HG increases TLR4‐MD2 interaction, as well as TLR4 interaction with MyD88 and TRIF (Figure 3F,G). Sch B pretreatment reduced the levels of MyD88‐TLR4 interaction, but had no effect on MD2‐TLR4 and TLR4‐TRIF (Figure 3F,G). We know that MyD88 also serves as an adapter protein for other TLRs, such as TLR2.^[^ ^14^ ^]^ Indeed, analysis of H9C2 cells exposed to HG showed the TLR2‐MyD88 interaction, which was reduced when cells were pretreated with Sch B (Figure S5, Supporting Information). These studies suggested that Sch B specifically targets MyD88.

### 2.4 Sch B Directly Binds to MyD88 to Inhibit its Activity

**Figure 4:**
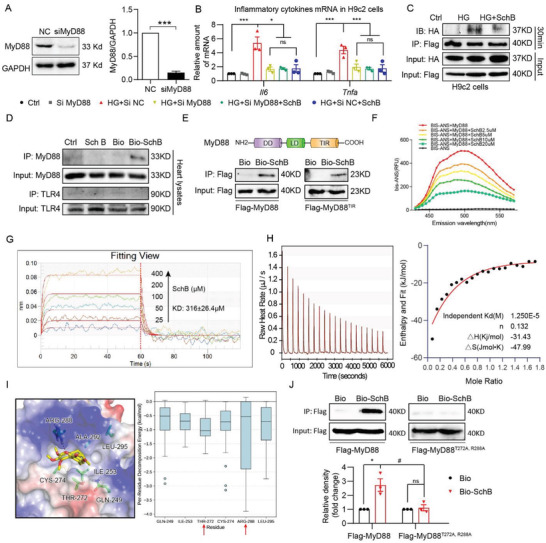
Sch B directly binds to MyD88. A) Western blot analysis of MyD88 knockdown in H9C2 cells. Cells were transfected with negative control siRNA (NC) or siRNA against MyD88 (siMyD88). GAPDH was used as loading control. (Values represent the mean ± SEM; *n* = 3; ***p < 0.001 compared to NC by unpaired two‐tailed Student's *t*‐test). B) qPCR analysis of inflammatory cytokines in H9C2 cells transfected with MyD88 siRNA. Transfected cells were treated with 10 × 10^−6^ m Sch B for 1 h before exposure to HG for 8 h. Untransfected (Ctrl) and cells transfected with negative control siRNA (NC) were used as control (values represent mean ± SEM; *n* = 3; ns = not significant; *p < 0.05; ***p < 0.001 compared to control, and #*p* < 0.05 compared to HG+siNC by unpaired two‐tailed Student's *t*‐test). C) H9C2 cells were transfected with Flag‐ and HA‐tagged MyD88. Cells were treated with 10 × 10^−6^ m Sch B for 1 h and then exposed to HG for 30 min. Flag was immunoprecipitated and HA was detected to examine MyD88 dimerization. D) Binding of biotinylated‐Sch B to MyD88 was determined by immunoblotting. Bio‐Sch B was added to streptavidin‐agarose beads. Untreated beads, unconjugated Sch B, and biotin alone were used as control. Lysates prepared from control mouse heart tissues were added. E) Western blot analysis of the binding of Bio‐Sch B to MyD88‐TIR domain. Lysates prepared from HEK 293T cells transfected with Flag‐tagged full MyD88 or Flag‐tagged MyD88 TIR domain only were incubated with Bio‐Sch B‐loaded beads or biotin‐loaded beads. Upper panel shows the structure of MyD88 and lower panel shows pulled proteins. F) Fluorescence spectroscopy utilizing bis‐ANS showing the binding of Sch B to rhMyD88. G) SPR analysis showing interaction between Sch B and recombinant MyD88 protein. Sch B was added at different concentrations and KD values were calculated (shown in the insert). H) ITC analysis for MyD88 binding to Sch B. Representative image shown. The left panel shows the representative titration thermograms, and the right panel shows the data integration with fitted curves (independent model) of Sch B with MyD88(TIR). I) The binding pocket of Sch B and the key residues with the lowest binding energy. Left panel shows the carbon atoms of six key residues’ side chain and Sch B represented as green sticks and yellow sticks, respectively. Right panel shows the boxplot of the per‐residue decomposition energy of the six residues. Red arrows indicate potentially important residues. J) Pull‐down analysis of the binding of Bio‐Sch B to mutant MyD88 containing T272A and R288A. HEK 393T cells were transfected with wildtype MyD88 or mutant variants. Lysates were used to detect binding to bio‐Sch B (mean ± SEM; *n* = 3; ns = not significant; *p < 0.05 compared to Bio; #*p* < 0.05 compared to Bio‐Sch B by unpaired two‐tailed Student's *t*‐test).

Following our discovery of MyD88 as the likely target of Sch B in cardiac cells, we wanted to examine exactly how this is achieved. We knocked down MyD88 in H9C2 cells and noted that it mimicked Sch B pretreatment, in terms of suppressing HG‐induced *Il6* and *Tnfa*, while no additive effects were seen when MyD88‐knockdown cells were pretreated with Sch B (**Figure** **4**A,B). Sch B also significantly reversed HG‐induced *Il6* and *Tnfa* gene transcription in H9C2 cells with MyD88 overexpression (Figure S6, Supporting Information). These results indicated that Sch B inhibited MyD88‐mediated inflammation. Since homodimerization of MyD88 is critically important for signaling,^[^ ^15^ ^]^ we transfected H9C2 cells with flag‐ and HA‐tagged MyD88 and observed that MyD88 dimerization is inhibited by Sch B (Figure 4C and Figure S7, Supporting Information). These data suggest that Sch B may directly bind or target MyD88. We then performed a simple pull‐down assay. We biotinylated Sch B (Figure S8A, Supporting Information), confirmed that it retained its biological activity by probing for inflammatory factor suppression (Figure S8B, Supporting Information), and then used it to examine interacting proteins. Application of mouse heart tissue lysate as the protein source showed Sch B interaction with MyD88 but not TLR4 (Figure 4D and Figure S9A, Supporting Information). Since all of reported small‐molecule MyD88 inhibitors bound to the TIR domain of MyD88, we suspected that this domain was the likely site of Sch B interaction. To test this experimentally, we transfected HEK 293T cells with either full‐length MyD88 or just the TIR domain of MyD88. Immunoprecipitation pull‐down confirmed that biotinylated Sch B interacts with the TIR domain of MyD88 (Figure 4E and Figure S9B, Supporting Information).

We next used additional assays to confirm the direct interaction between Sch B and MyD88. We found that Sch B dose‐dependently reduced the fluorescence signal of rhMyD88 protein and bis‐ANS fluorescence probe,^[^ ^16^ ^]^ signifying a competitive interaction (Figure 4F). Surface plasmon resonance (SPR) showed that Sch B interacts with rhMyD88 with an association rate constant (KD) of ≈316 × 10^−6^ m (Figure 4G). Similar analysis with rhTLR4 showed no interaction between Sch B and TLR4 (Figure S10, Supporting Information). Lastly, we performed isothermal titration calorimetry (ITC) assay and show that Sch B interacts with rhMyD88 with a binding Kd of 125 × 10^−6^ m (Figure 4H). We then proceeded with examining where in the TIR domain of MyD88 would Sch B interact. To do this, we utilized the MyD88 TIR crystal structure to perform global docking. Four potential binding sites emerged (Figure S11A,B). Of these, site 3 showed the lowest binding free energy and was selected for finer, key residue identification. A per‐reside energy decomposition calculation was performed for the 27 docking poses which bound to site 3. We identified six residues with the lowest average energy: Gln‐249, Ile‐253, Thr‐272, Cys‐274, Arg‐288, and Leu‐295 (Figure 4I). Of these, we selected Thr‐272 and Arg‐288 for further study, based on their energy values. We transfected HEK 293T cells with either wildtype MyD88 or mutant MyD88 with T272A and R288A. Biotinylated Sch B pulled‐down wildtype MyD88 but was unable to interact with mutant MyD88 (Figure 4J). These results suggest that Thr‐272 and Arg‐288 in the TIR domain may interact with Sch B.

### 2.5 Sch B Protects Heart Tissues in Type 2 Diabetic Mice through MyD88 Inhibition

**Figure 5:**
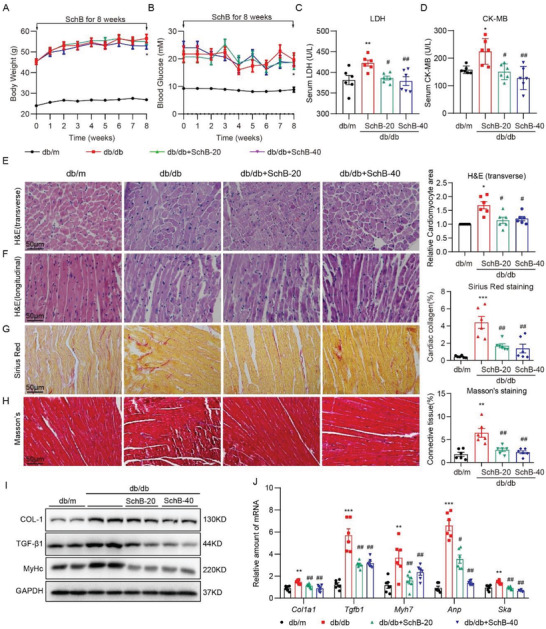
Sch B suppresses cardiac hypertrophy and fibrosis in *db/db* mice. A) Body weight of mice at time points indicated. B) Blood glucose levels in mice. C,D) Levels of serum C) LDH and D) CK‐MB in mice. E,F) Representative H&E‐stained images of mouse heart tissues showing E) transverse and F) longitudinal sections. Right panel showing quantification of cardiomyocyte size determined from transverse sections (scale bar = 50 µm). G) Representative images of Sirius Red staining of heart tissues. Quantification of collagen levels is shown on right (scale bar = 50 µm). H) Representative images of Masson's trichrome staining of heart tissues. Quantification of fibrosis is shown on right (scale bar = 50 µm). I) Immunoblotting of Col‐1, TGF‐*β*1, *β*‐MyHc in heart tissue. GAPDH was used as loading control. J) qPCR analysis of cardiac fibrosis and hypertrophy genes in heart tissues. mRNA levels were normalized to *Actb*. Data in all panels are shown as mean ± SEM (*n* = 6 per group; *p < 0.05, **p < 0.01, ***p < 0.001 compared to *db/m* and #*p* < 0.05, ##*p* < 0.01 compared to *db/db* by one‐way ANOVA followed by Bonferroni's multiple comparisons test).

Based on the promising in vitro protective activity of Sch B on HG‐challenged cardiac cells, we wanted to determine whether such protection is replicated in mouse models of diabetes. We utilized *db/db* mice treated with Sch B to achieve this goal. Heterozygous db/m mice served as nondiabetic controls. Previous studies have used 20–80 mg kg^−1^ Sch B in models of heart dysfunction and shown significant effects.^[^ ^11^, ^17^ ^]^ Diabetic (*db/db*) mice were treated with either 20 or 40 mg kg^−1^ Sch B for 8 weeks. In our study, Sch B did not change the body weight gain or blood glucose levels in mice (**Figure** **5**A,B). However, serum markers of cellular damage, lactate dehydrogenase (LDH) and creatine kinase‐cardiac (CK‐MB), did not show the same increase as seen in untreated *db/db* mice (Figure 5C,D), suggesting that Sch B treatment prevented heart damage. Assessment of heart function in mice using echocardiography showed that Sch B afforded protection in both systolic and diastolic function (**Table** **1** and Figure S12, Supporting Information). Analysis of the harvested heart tissues showed structural and hypertrophic changes in *db/db* mice but not mice treated with Sch B (Figure 5E,F). Sirius red and Masson's trichrome staining also showed suppressive activity of Sch B on fibrotic responses in *db/db* mice (Figure 5G,H). We confirmed these histological analyses by probing for select gene and protein markers of hypertrophy and fibrosis (Figure 5I,J). These studies show that Sch B protects *db/db* diabetic mice from cardiac dysfunction and structural alterations.

**Table 1:** Echocardiographic parameters in *db/db* mice

	*db/m* (*n* = 6)	*db/db*		
		Vehicle (*n* = 6)	Sch B‐20 (*n* = 6)	Sch B‐40 (*n* = 6)
EF %^a)^	84.24 ± 1.38	72.33 ± 1.73***	80.24 ± 1.95^##^	78.89 ± 0.98^#^
FS %	47.05 ± 1.68	35.8 ± 1.3***	42.99 ± 1.93^#^	41.7 ± 0.85^#^
LVIDd (mm)	2.32 ± 0.07	2.85 ± 0.12**	2.75 ± 0.13	2.58 ± 0.11
LVIDs (mm)	1.35 ± 0.06	1.83 ± 0.1**	1.57 ± 0.09	1.53 ± 0.09
LVFWd (mm)	0.62 ± 0.02	0.72 ± 0.02**	0.69 ± 0.01	0.67 ± 0.02
LVFWs (mm)	0.92 ± 0.02	0.93 ± 0.02	0.85 ± 0.04	0.87 ± 0.02
LVPWd (mm)	0.6 ± 0.02	0.73 ± 0.02**	0.7 ± 0.02	0.68 ± 0.02
LVPWs (mm)	0.82 ± 0.03	0.9 ± 0.02	0.87 ± 0.02	0.9 ± 0.02
IVSd (mm)	0.62 ± 0.02	0.75 ± 0.02**	0.7 ± 0.02	0.7 ± 0.02
E wave (m s^−1^)	0.79 ± 0.08	0.6 ± 0.03*	0.61 ± 0.04	0.63 ± 0.03
IRT (ms)	18 ± 0.62	25 ± 1.56***	19.33 ± 0.73^##^	22 ± 0.78
HW/TL	8.29 ± 0.42	8.74 ± 0.3	7.76 ± 0.27	8.44 ± 0.3

^a)^ EF = ejection fraction; FS = fractional shortening; LVIDd/s = diastole/systole left ventricle internal dimension; LVFWd/s = LV free wall thickness at diastole/systole; LVPWd/s = LV posterior wall thickness at diastole/systole; IVSd = diastole interventricular septal thickness; IRT = isovolumic relaxation time; HW/TL, heart weight/tibia length. Data presented as mean ± SEM; *p < 0.05, **p < 0.01, ***p < 0.001 compared to db/m; #*p* < 0.05, ##*p* < 0.01 compared to db/db.

**Figure 6:**
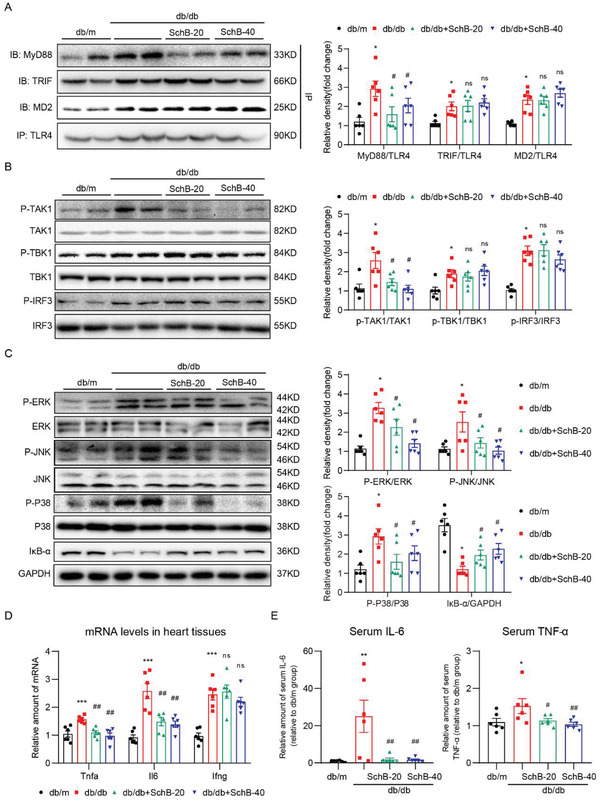
Sch B reduces inflammatory responses in *db/db* mice by inhibiting MyD88. A) Co‐immunoprecipitation showing formation of MD2/TLR4, TLR4/MyD88, and TLR4/TRIF complexes in heart tissues of *db/db* mice. TLR4 was immunoprecipitated (IP) and levels of MD2, TRIF, and MyD88 were detected by immunoblotting (IB). Densitometric quantification is shown on right. B) Western blot analysis of pathway activation downstream of MyD88 in heart tissues. Phosphorylated TAK1, TBK1, and IRF3 were detected. Total proteins were used as control. Densitometric quantification shown on right. C) Analysis of MAPK and NF‐*κ*B activation in heart tissues of *db/db* mice. Phosphorylated ERK, JNK, and p38 were detected. Total proteins were used as control. I*κ*B‐*α* was used to measure NF‐*κ*B activity, with GAPDH as the loading control. Right panel shows densitometric quantification. D) mRNA levels of *Tnf*, *Il6*, and *Ifng* in heart tissues of mice. Transcripts were normalized to *Actb*. E) Protein levels of IL‐6 and TNF‐*α* in serum of *db/db* mice were determined by enzyme‐linked immunoassay (ELISA). Data in all panels are shown as mean ± SEM (*n* = 6 per group; *p < 0.05, **p < 0.01, ***p < 0.001 compared to *db/m*; #*p* < 0.05, ##*p* < 0.01, ns = not significant compared to *db/db* by one‐way ANOVA followed by Bonferroni's multiple comparisons test).

Since our in vitro studies pointed to the modulation of MyD88‐dependent inflammatory responses by Sch B, we proceeded to examine heart tissues of mice for such mechanism. Immunoprecipitation of TLR4 from heart tissue lysates showed increased levels of associated MyD88, MD2, and TRIF in *db/db* mice (**Figure** **6**A). Sch B treatment decreased the interaction between TLR4 and MyD88 but had no effect of MD2 or TRIF associated with TLR4. Analysis of downstream signaling hubs, TAK1, TBK1, and IRF3, confirmed that Sch B primarily modulated the MyD88‐dependent pathway (Figure 6B). As expected, both MAPK and NF‐*κ*B activation in *db/db* mouse hearts was suppressed by Sch B treatment (Figure 6C and Figure S13, Supporting Information). We then assayed for MyD88 target genes. The mRNA levels of *Tnfa* and *Il6* (MyD88 pathway) and *Ifng* (TRIF pathway) were induced in heart tissues of *db/db* mice, and only *Tnf* and *Il6* induction was blocked by Sch B (Figure 6D). Measuring the serum levels of TNF and IL‐6 in these mice confirmed the gene expression data (Figure 6E). Cardiac macrophage infiltration, induced by increased levels of various proinflammatory cytokine, chemokines, and adhesion molecules in heart, is also an important phenotype of cardiac inflammatory responses in DCM. We further examined the effect of Sch B on cardiac macrophage infiltration. F4/80 immunostaining assay indicated that Sch B treatment significantly reduced diabetes‐induced macrophage infiltration in mouse hearts (Figure S14, Supporting Information). Overall, our data in the *db/db* model showed that Sch B protects the heart tissues by inhibiting MyD88‐mediated inflammatory responses.

### 2.6 Sch B Prevents DCM in Type 1 Diabetic Mice by Inhibiting MyD88

**Figure 7:**
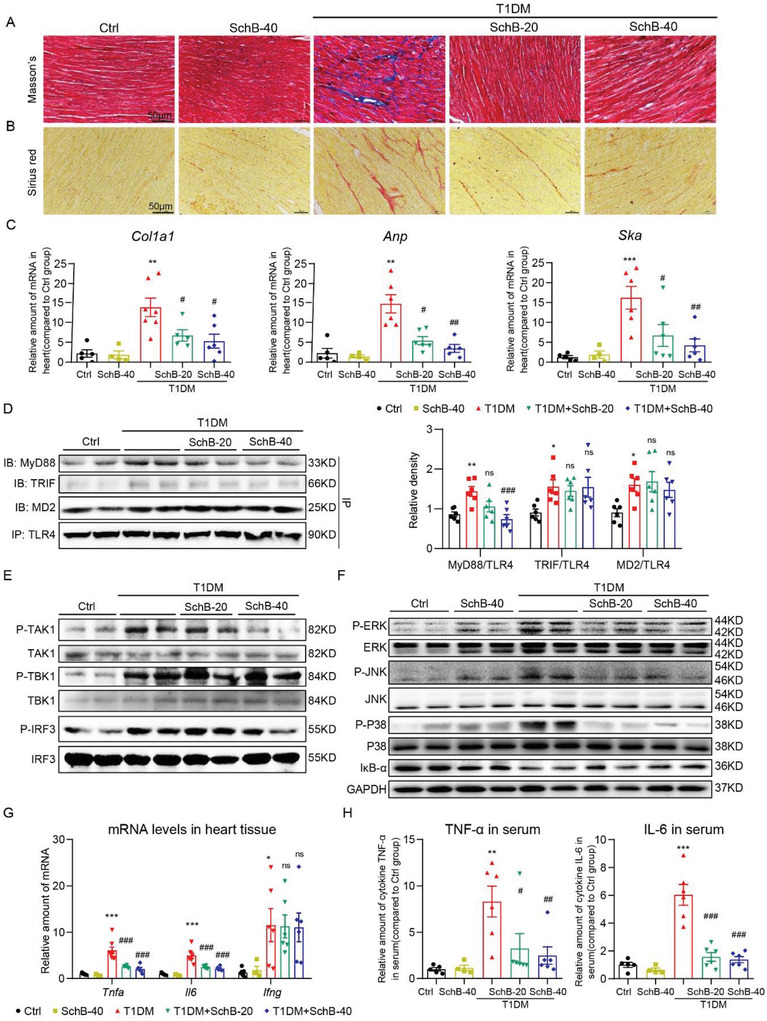
Sch B treatment suppresses cardiac injury in STZ‐induced model of type 1 diabetes. A,B) Representative images of A) Masson's trichrome and B) Sirius red staining of heart tissues (scale bar = 50 µm). C) mRNA levels of *Cola1*, *Anp*, and *Ska* in heart tissues. Transcripts were normalized to *Actb*. D) TLR4 was immunoprecipitated (IP) from mouse heart lysates and MD2, TRIF, and MyD88 were detected by immunoblotting (IB). Right panel shows the densitometric quantification. E) Western blot analysis of pathway activation downstream of MyD88 in heart tissues. Phosphorylated TAK1, TBK1, and IRF3 were detected. Total proteins were used as control. F) Analysis of MAPK and NF‐*κ*B activation in heart tissues of STZ‐induced T1DM mice. Phosphorylated ERK, JNK, and p38 were detected. Total proteins were used as control. I*κ*B‐*α* was used to measure NF‐*κ*B activity, with GAPDH as the loading control. G) mRNA levels of *Tnfa*, *Il6*, and *Ifng* in heart tissues of mice. Transcripts were normalized to *Actb*. H) Protein levels of IL‐6 and TNF‐*α* in serum of mice were examined by ELISA. Data in panels (C), (D), (G), and (H) are shown as mean ± SEM (*n* = 6 per group; *p < 0.05, **p < 0.01, ***p < 0.001 compared to Ctrl; ns = not significant, #*p* < 0.05, ##*p* < 0.01, ###*p* < 0.001 compared to T1DM by one‐way ANOVA followed by Bonferroni's multiple comparisons test).

We also performed the same studies in a type 1 model of diabetes. We induced diabetes in C57BL/6 mice using streptozotocin (STZ) and treated the mice with Sch B for 16 weeks. Similar to our results in *db/db* mice, Sch B did not affect blood glucose levels or body weight gain in mice (Figure S15A,B, Supporting Information). Assessment of heart function revealed a similar protection by Sch B as seen in the *db/db* model (**Table** **2**). Histologically, reduced fibrosis was seen in heart tissues of diabetic mice treated with Sch B as compared to untreated diabetic mice (**Figure** **7**A,B and Figure S15C,D, Supporting Information). These structural alterations were confirmed by gene markers of hypertrophy and fibrosis (Figure 7C). Specifically, Sch B treatment of diabetic mice prevented TLR4‐MyD88 interaction (Figure 7D), activation of TAK1 but not TBK1 or IRF3 (Figure 7E and Figure S16A, Supporting Information), activation of MAPKs and NF‐*κ*B (Figure 7F and Figures S16B and S17, Supporting Information), expression of *Tnf* and *Il6* (Figure 7G), and elevation of serum TNF and IL‐6 (Figure 7H). F4/80 immunostaining assay also showed reduced macrophage infiltration in the hearts of Sch B‐treated diabetic mice (Figure S18, Supporting Information). Collectively, our animal modeling studies showed that Sch B reduces indices of DCM in both type 1 and type 2 diabetic models. Importantly, the studies point to MyD88 as a common pathogenetic mediator of inflammatory injury in both types of diabetes.

**Table 2:** Echocardiographic parameters in streptozotocin‐induced diabetic model

	Ctrl (*n* = 6)	Sch B‐40 (*n* = 6)	T1DM		
			Vehicle (*n* = 6)	SchB‐20 (*n* = 6)	Sch B‐40 (*n* = 6)
EF %^a)^	81.9 ± 0.869	80.3 ± 1.269	73.7 ± 0.876***	79.0 ± 0.705^###^	79.2 ± 0.767^###^
FS %	43.1 ± 1.623	42.1 ± 1.632	35.1 ± 0.693***	40.3 ± 0.688^##^	40.2 ± 0.896^##^
LVIDd (mm)	2.20 ± 0.041	2.16 ± 0.040	2.35 ± 0.056*	2.22 ± 0.049	2.16 ± 0.042^#^
LVIDs (mm)	1.26 ± 0.042	1.30 ± 0.037	1.47 ± 0.064**	1.35 ± 0.022	1.30 ± 0.026^#^
LVFWd (mm)	0.73 ± 0.004	0.72 ± 0.019	0.77 ± 0.005**	0.75 ± 0.009	0.73 ± 0.005^#^
LVFWs (mm)	0.86 ± 0.002	0.86 ± 0.002	0.84 ± 0.009	0.84 ± 0.011	0.84 ± 0.011
LVPWd (mm)	0.71 ± 0.004	0.71 ± 0.012	0.75 ± 0.008**	0.72 ± 0.004^#^	0.72 ± 0.008^#^
LVPWs (mm)	0.87 ± 0.008	0.87 ± 0.006	0.89 ± 0.004*	0.89 ± 0.003	0.86 ± 0.008^#^
IVSd (mm)	0.68 ± 0.002	0.69 ± 0.009	0.74 ± 0.011***	0.71 ± 0.006^#^	0.70 ± 0.007^#^
IVSs (mm)	0.86 ± 0.009	0.86 ± 0.005	0.87 ± 0.008	0.86 ± 0.008	0.84 ± 0.009
Heart weight, mg	133.5 ± 4.60	134.5 ± 5.81	104.1 ± 4.93***	107.1 ± 5.53^#^	103.6 ± 6.78^##^
HW/BW, mg g^−1^	4.58 ± 0.08	4.72 ± 0.1	5.32 ± 0.07***	4.90 ± 0.17^#^	4.85 ± 0.11^#^

^a)^ EF = ejection fraction; FS = fractional shortening; LVIDd/s = diastole/systole left ventricle internal dimension; LVFWd/s = LV free wall thickness at diastole/systole; LVPWd/s = LV posterior wall thickness at diastole/systole; IVSd/s = diastole/systole interventricular septal thickness; IRT = isovolumic relaxation time; HW/BW = heart weight/body weight. Data presented as mean ± SEM; *p < 0.05, **p < 0.01, ***p < 0.001 compared to Ctrl; #*p* < 0.05, ##*p* < 0.01, ###*p* < 0.001 compared to T1DM.

### 2.7 Cardiomyocyte‐Specific MyD88 Knockout Prevented STZ‐Induced Cardiac Inflammation and Injury

**Figure 8:**
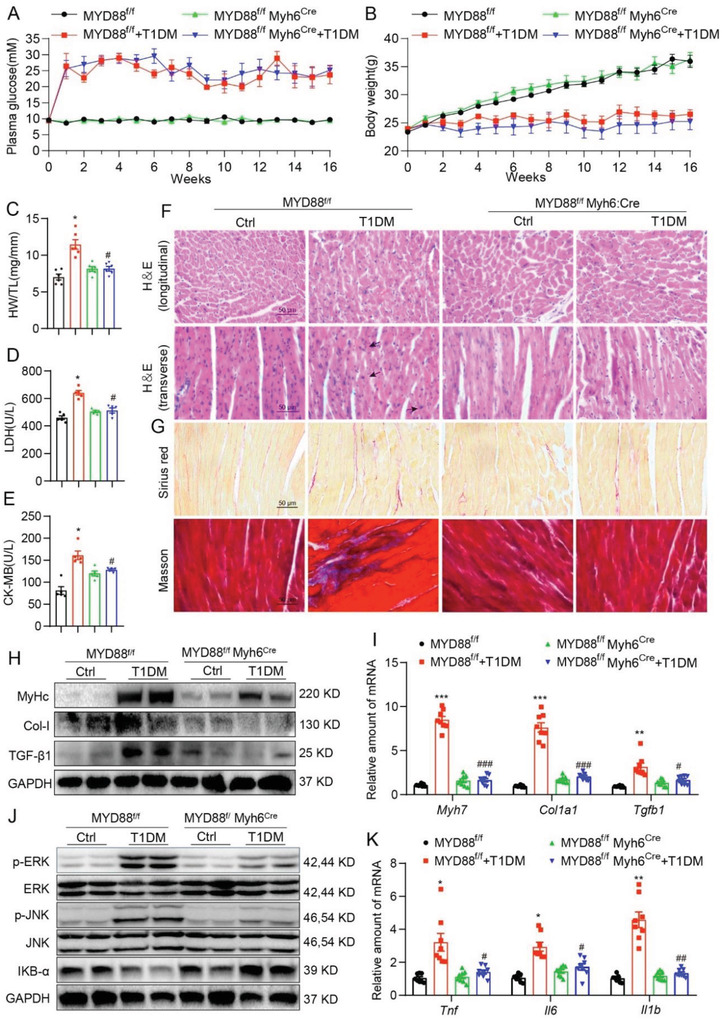
Cardiomyocyte‐specific MyD88 knockout prevents STZ‐induced cardiac inflammation and injury. Male MyD88^f/f^ and MyD88^f/f^Myh6^Cre^ mice were injected with STZ to induce T1DM. A) Fasting blood glucose and B) body weight were recorded weekly for 16 weeks. Heart tissue and blood samples were collected at termination for the determination of C) heart weight/tibia length (HW/TL) ratios, D) serum LDH, and E) serum CK‐MB activity. Representative images (400X magnification) of F) H&E staining (longitudinal and transverse), G) Sirius red and Masson's trichrome staining of the heart tissues were shown (scale bar = 50 µm). Representative H) western blot and I) qPCR analysis of MyHc, Col‐1, and TGF‐*β* in the heart tissue were shown. J) Levels of p‐ERK/ERK, p‐JNK/JNK, and I*κ*B‐*α* were determined by western blot. GAPDH was loading control. K) qPCR analyses of *Tnf*, *Il6*, and *Il1b* in the heart tissues were shown. All data are shown as mean ± SEM (*n* = 6 per group; *p < 0.05, **p < 0.01, ***p < 0.001 compared to MyD88^f/f^; ns = not significant, #*p* < 0.05, ##*p* < 0.01, ###*p* < 0.001 compared to MyD88^f/f^+T1DM by one‐way ANOVA followed by Bonferroni's multiple comparisons test).

Finally, we explored the protective effect of Sch B in targeting MyD88 using cardiomyocyte‐specific MyD88‐knockout mice. Immunofluorescence double‐staining in mouse heart sections confirmed the expression and localization of MyD88 mainly in the cardiomyocytes of diabetic mice (Figure S19A–C, Supporting Information), which was further validated by measuring MyD88 expression in rat primary cardiomyocytes and cardiac fibroblasts via immunoblotting (Figure S19D, Supporting Information). Then, the cardiomyocyte‐specific MyD88‐knockout mice (MyD88^f/f^Myh6^Cre^) were generated by crossing Myh6^Cre^ and MyD88^f/f^ mice (Figure S20A–C, Supporting Information). Cardiac MyD88 deletion did not affect hyperglycemia and body weight loss induced by STZ (**Figure** **8**A,B). As expected, cardiac MyD88 deletion significantly prevented STZ‐increased heart‐weight/body‐weight (HW/BW) ratio and plasma LDH and CK‐MB levels (Figure 8C–E). Hematoxylin and eosin (H&E) staining showed structural abnormalities and increased cardiomyocyte area were completely prevented in STZ‐treated MyD88‐deficient mice (Figure 8F and Figure S21A, Supporting Information). Masson's trichrome and Sirius red staining analysis showed that increased collagen deposition in the hearts was also prevented in MyD88^f/f^‐Myh6^Cre^ mice (Figure 8G and Figure S21B,C, Supporting Information), which was confirmed by gene markers of hypertrophy and fibrosis (Figure 8H,I and Figure 21D, Supporting Information). We then examined MyD88‐dependent inflammatory cascade in mouse heart tissues, including MAPKs activation, I*κ*B‐*α* degradation, p65 nuclear translocation, and cytokine gene expression. As seen in Sch B‐treated type 1 diabetic mice, this cascade was markedly prevented by cardiac MyD88 gene knockout (Figure 8J,K and Figures S21E and S22, Supporting Information). These data validated cardiomyocyte MyD88 as the potential target for the treatment of DCM.

## 3 Discussion

Myocardial inflammation is a causative factor in diabetes‐induced cardiac dysfunction. In this study, we have shown that Sch B prevents HG‐induced hypertrophic and fibrotic responses in cardiomyocytes, possibly through selectively modulating inflammatory responses. We discovered that Sch B directly binds to MyD88 protein to prevent its association with TLRs, which then prevents MyD88‐dependent proinflammatory signaling pathway activation, expression of inflammatory cytokines, and macrophage infiltration. Treatment of type 1 and type 2 diabetic mice with Sch B confirmed this cardioprotective property, and the same mechanisms in heart tissues were observed in our culture studies. Finally, the role of MyD88 in type 1 DCM was validated using cardiomyocyte‐specific MyD88‐knockout mice. These key findings are summarized in the Graphical Abstract.

Perhaps one of the most significant contributions of this study is the discovery of a natural product, Sch B, as a new MyD88 inhibitor. Administration of Sch B has been shown to produce desirable prevention of oxidative and inflammatory injury in models of cardiovascular diseases.^[^ ^18^ ^]^ Chen and colleagues showed that administering Sch B at 80 mg kg^−1^ daily in mice offered protection against myocardial infarction.^[^ ^19^ ^]^ Our study, together with published reports on Sch B in other systems, suggests that Sch B may have broad impact in cardiovascular diseases. However, the molecular mechanism and target of Sch B are still unknown which limits the application of Sch B. Our results from RNA‐seq and inflammatory RNA microarray showed the unique profile of cytokines being modulated following Sch B treatment of cardiac cells, pointing to the involvement of MyD88‐dependent TLR pathway. Indeed, a series of examinations in Figure 3 show that Sch B modulates the MyD88‐dependent pathway, while leaving the TRIF‐pathway intact. This specificity is afforded by the direct binding of Sch B to MyD88 in the TIR domain. These results point to MyD88 as a potential therapeutic target. Studies have shown that MyD88‐knockout mice lack inflammatory cytokine induction in response to TLR4 ligand lipopolysaccharide.^[^ ^20^ ^]^ In addition to TLR4, responses to TLR2 ligands are also abolished in mice lacking MyD88.^[^ ^21^ ^]^ Furthermore, signaling through TLR5/7/9 are blunted.^[^ ^22^ ^]^ Therefore, agents that target MyD88 would offer broad‐spectrum anti‐inflammatory activities. Sch B fits this profile. Given that MyD88 is a common adaptor in TLRs/IL‐1R‐mediated innate immunity and inflammation, the finding that Sch B directly targets MyD88 may provide the underlying explanation for the extensive biological activity of Sch B.

Unlike other signaling proteins in the TLR/IL‐1R pathway, there is scarcity in the range of available MyD88 inhibitors. Besides the typical gene silencing approaches, there are only a few peptides (e.g., ST2825) and small molecule inhibitors (e.g., T5996207 and TSI‐13‐57) that have shown to inhibit MyD88.^[^ ^23^ ^]^ Our study identified a natural compound Sch B that specifically binds to the MyD88 TIR domain. It is also important to note that the Thr‐272/Arg‐288 in TIR domain participate in Sch B‐MyD88 interaction, which will greatly contribute to the further design and development of new small‐molecule MyD88 inhibitors. The obvious next set of studies would include examining whether Sch B protects against other amplified MyD88 pathway conditions, biodistribution, and toxicity studies of Sch B in preclinical models, and discovery‐based identification of other Sch B‐interacting proteins.

Although several anti‐inflammatory medications have been reported to prevent or suppress DCM in preclinical disease models, the molecular mechanisms and targets are controversial.^[^ ^24^ ^]^ Activation of TLRs, especially TLR2 and TLR4, has been associated with increased susceptibility to diabetic vascular complications, nephropathy, cardiomyopathy, retinopathy, and neuropathy, by initiating the activation of proinflammatory signals.^[^ ^25^ ^]^ However, TLR4 antagonists could not block inflammation mediated by other TLRs, which may be the main reason for the failure of TLR4 antagonists, eritoran, and TAK‐242, in treating sepsis.^[^ ^26^ ^]^ Although MD2/TLR4 inhibitors have been reported to prevent DCM in experimental animals, we may consider that the inhibition of MyD88, a molecular target downstream to almost all TLRs and IL‐1R, may be a more appropriate strategy than antagonism of certain TLRs. MyD88‐deficient mice have been found to be unresponsive to endotoxin.^[^ ^20^ ^]^ Our group previously showed that MyD88 inhibitor attenuated obesity‐induced heart inflammation and injuries in mice.^[^ ^27^ ^]^ To the best of our knowledge, this is the first time that the evaluation of the cardioprotective effects of MyD88 inhibition against DCM in both T1D and T2D mice was conducted. Furthermore, cardiomyocyte‐specific MyD88 gene knockout also protects hearts in diabetic mice. It is interesting that neither genetic nor pharmacologic inhibition of MyD88 affected hyperglycemia in mice with T1D or T2D, indicating that the cardiac benefits observed in T1D and T2D mice were mainly due to the anti‐inflammatory effect of MyD88 inhibition. In addition, the systemic MyD88‐knockout mice have been found to be viable without any overt phenotype.^[^ ^20^ ^]^ Our study also showed good efficacy and high safety of Sch B in both DCM models. These data provide full evidence for MyD88 as a potential target for the treatment of DCM.

A limitation of this study may be the absence of the evaluation of MyD88 in macrophages in the heart. MyD88 is originally a key protein in macrophage innate immunity. As we know, both cardiomyocytes and macrophages contribute to inflammatory DCM. Our group have reported that MD2‐TLR4/MyD88 signaling pathway in both cardiomyocytes and macrophages mediates hyperglycemia‐induced cardiac inflammation and plays an important role in the development of DCM.^[^ ^4b^ ^]^ Although our study focuses on Sch B targeting MyD88 and inflammation in cardiomyocytes, future studies should examine whether MyD88 in macrophages contributes to inflammatory DCM and mediates the cardioprotective effects of Sch B. In addition, although we have validated the role of MyD88 in mediating the bioactivity of Sch B in cultured cardiomyocytes, where knocking down MyD88 blocked anti‐inflammatory effects of Sch B, it may strengthen our conclusion to evaluate the cardioprotective effects of Sch B in MyD88‐knockout mice with diabetes.

A surprising finding in our study is that Sch B did not alter the TRIF‐mediated pathway in both diabetic models. To the best of our knowledge, there are no studies that report the functional significance of MyD88 and TRIF pathways in DCM. One possible explanation could be that the TRIF pathway is not involved in diabetes‐induced inflammatory cytokine production, fibrosis, or hypertrophy in the heart. It is also possible that TRIF is a minor player in the activation of MAPK/NF‐*κ*B pathway^[^ ^28^ ^]^ and that MyD88 inhibition is sufficient for protection. It would be important to determine the exact contribution of TRIF‐driven IFNs, although our data showed that inhibition of MyD88 arm of the TLR pathway with Sch B, or MyD88 gene knockout, almost completely prevented cardiomyopathy. In support of such an approach, a recent study has shown that unlike TLR2/4‐MyD88 signaling, lack of TRIF had no impact on myocardial cytokines or neutrophil recruitment after ischemia/reperfusion, but myocardial apoptosis was significantly reduced in *Trif* knockout mice.^[^ ^29^ ^]^ Therefore, determining whether TRIF inhibition offers added benefit in diabetes would be important in the future.

## 4 Conclusion

In summary, we have shown that diabetes increases the interaction between MyD88 and TLR4 to initiate inflammatory injury in cardiomyocytes. This injury is mediated through production of proinflammatory cytokines and activation of TAK1‐MAPK/NF‐*κ*B. These events result in hypertrophic and fibrotic changes in heart tissues of mice and in isolated cardiac cells. Sch B directly binds to MyD88 TIR domain and inhibits MyD88 activity by blocking the homodimerization of MyD88 TIR domain. The direct targeting and inhibition of Sch B on MyD88 lead to subsequent suppression of MyD88‐dependent downstream signaling pathways including TBK1/IRF3 and TAK1/MAPKs/NF‐*κ*B and inflammatory responses in cardiomyocytes. Either inhibition of MyD88 by Sch B or cardiomyocyte‐specific MyD88 deficiency dampens the proinflammatory milieu and prevents DCM in diabetic mice. These findings uncovered the direct target of Sch B and highlight the importance of MyD88 as a key pathogenic factor in DCM. Furthermore, we provided support for future research and the potential use of Sch B for the treatment of DCM.

## 5 Experimental Section

### General Reagents

Sch B (high performance liquid chromatography purity > 98%, Figure S1A, Supporting Information) and L‐glucose were purchased from Sigma‐Aldrich (St. Louis, MO). Sch B was dissolved in dimethyl sulfoxide (DMSO) for in vitro experiments and in 1% sodium carboxymethyl cellulose (CMC‐Na) solution for in vivo studies. Recombinant human MyD88 (rhMyD88) was purchased from R&D Systems (Minneapolis, MN, USA). Antibodies against Collagen 1 (Col‐1), transforming growth factor‐*β*1 (TGF‐*β*1), myosin heavy chain beta isoform (*β*‐MyHc), MyD88, TRIF, and MD2 were purchased from Abcam (Waltham, MA). Antibodies against phosphorylated (p‐) and/or total proteins, including inhibitor of *κ*B‐*α* (I*κ*B‐*α*), p65 subunit of NF‐*κ*B, Lamin B, TANK binding protein (TBK1), TGF*β*‐activated kinase‐1 (TAK1), interferon response factor‐3 (IRF3), extracellular signal‐regulated kinase (ERK), Junas N‐terminal kinase (JNK), p38, and GAPDH were purchased from Cell Signaling Technology (Danvers, MA). Antibodies against TLR4, HA, Flag tag, and horseradish peroxidase‐conjugated secondary antibodies were purchased from Santa Cruz Biotechnology (Santa Cruz, CA). Rhodamine phalloidin was purchased from Enzo Life Sciences (Farmingdale, NY). FLAG‐tagged MyD88(FL) and FLAG‐tagged MyD88(TIR) plasmids were constructed and sequenced by Shanghai GeneChem Co (Shanghai, China). Cytosolic and nuclear fraction extraction kit was obtained from Beyotime Biotech (China).

### Cardiomyocyte Cultures

Embryonic rat heart‐derived cardiomyocyte‐like H9C2 cells were obtained from the Shanghai Institute of Biochemistry and Cell Biology (Shanghai, China), and cultured in Dulbecco's modified Eagle medium (DMEM) medium (Gibco, Eggenstein, Germany) containing 1000 mg L^−1^ of D‐glucose. The media was supplemented with 10% fetal bovine serum, and 100 U mL^−1^ of penicillin and streptomycin. Where indicated, media containing HG comprised of 33 mmol L^−1^ glucose. Previous study already excluded the osmotic effect of HG using 33 × 10^−3^ m mannitol.^[^ ^4b^ ^]^

Primary cultures of cardiac myocytes and fibroblasts were prepared from the ventricles of neonatal Sprague–Dawley rats (Wenzhou Medical University Animal Centre, Wenzhou, China) as described previously.^[^ ^30^ ^]^ Human embryonic kidney (HEK) 293T cells were obtained from the Shanghai Institute of Biochemistry and Cell Biology and cultured in DMEM medium containing 1000 mg L^−1^ of D‐glucose.

### RNA‐seq and Pathway Enrich Analysis

Total RNA was extracted from H9C2 cells using the TRIzol (Takara). Cells were pretreated with 10 × 10^−6^ m Sch B for 1 h before exposure to 33 × 10^−3^ m glucose for 8 h. Construction of RNA‐seq library and RNA‐seq was completed by BGI Wuhan Genomics Institute using the BGISEQ‐500 platform.^[^ ^31^ ^]^ Briefly, samples were enriched by oligo‐dT selection, purified, and fragmented, and reverse‐transcribed into cDNA which was then end‐repaired and 3′‐adenylated. This was followed by adaptor ligation. Ligation products were purified and PCR‐amplified using PCR primer fragments. PCR products were then heat‐denatured, and ssDNA was cyclized by splint‐oligo and DNA ligase. Finally, the prepared library was sequenced. Clean reads were aligned to rat reference genome RGSC 6.0/rn6 by HISAT. RSEM was used to calculate gene expression, and differential expression was determined by BGI algorithm. Differentially expressed gene analysis was performed using DESeq2 (version 1.4.5) R package,^[^ ^32^ ^]^ with fold change > = 2 or < = 0.05, and adjusted *p* value < 0.05. Pathway enrichment analysis of the differentially expressed genes was performed using the Wikipathway 2021 analysis in modEnrichr suite (https://www.maayanlab.cloud/modEnrichr/).

### Immunofluorescence Cell Staining

Cells were fixed with 4% paraformaldehyde, permeabilized with 0.1% Triton X‐100, and stained with p65 antibody at 1:200 dilution overnight at 4 °C. Phycoerythrin (PE)‐conjugated secondary antibody (1:200) was applied for detection. Cells were counterstained with 4′,6‐diamidino‐2‐phenylindole (DAPI) nuclear stain. Images for NF‐*κ*B P65 were captured using an epifluorescence microscope equipped with a digital camera (Nikon, Tokyo, Japan).

### MyD88 Expression and Knockdown

MyD88 gene silencing in cells was achieved by transfecting cells with siRNA (5’‐GACAGACUAUCGGCUUAAATT‐3’) using LipofectAMINE 2000 (Invitrogen, Carlsbad, CA). Knockdown was verified by western blotting. Expression of total MyD88, TIR domain of MyD88, and mutant MyD88 (T272 or R288) was achieved by transfecting cells with the encoding plasmids under LipofectAMINE 2000. Expression was verified by western blotting.

### Determination of Cytokine Levels

Tumor necrosis factor‐*α* (TNF‐*α*) and interleukin‐6 (IL‐6) proteins in culture media and serum samples were determined by using cytokine‐specific ELISA kits (eBioscience, San Diego, CA). The amount of TNF‐*α* and IL‐6 in samples was normalized to total proteins.

### MyD88‐Sch B Binding Assays

Binding of Sch B to MyD88 was determined by multiple assays: SPR, ITC, pull‐down assays, and Bis‐ANS fluorescence dye (4,4′‐dianilino‐1,1′‐binaphthyl‐5,5′‐disulfonic acid dipotassium salt).

For SPR, kinetics of rhMyD88 or rhTLR4 binding to Sch B were assessed using BioLayer interferometry with an OctetK2 system (ForteBio Inc., Menlo Park, CA). Different concentrations of Sch B (400 × 10^−6^, 200 × 10^−6^, 100 × 10^−6^, 50 × 10^−6^, 25 × 10^−6^, and 0 × 10^−6^ m) were prepared with running buffer [PBST (0.1%Tween 20), pH 7.4, and 8% DMSO]. Super‐streptavidin biosensors (Forte´Bio Inc., Menlo Park, CA) were used to capture rhMyD88 or rhTLR4, biotinylated with NHS‐PEO4‐biotin (Pierce), onto the surface of the sensor. After reaching baseline, sensors were moved to association step for 60 s and then dissociated for 60 s. Curves were corrected by a double‐referencing technique, using both Super‐streptavidin pins dipped into the experimental wells and a buffer‐only reference. After double referencing corrections, subtracted binding interference data were applied to the calculations of binding constants using the FortéBio analysis software (Version: 9.0.0.10) provided with the instrument.

For ITC, potential interactions between Sch B and rhMyD88 were determined using the Nano‐ITC instrument (TA instruments, New Castle, DE) at 25 °C. rhMyD88(TIR) and Sch B were dissolved in phosphate‐buffered saline (PBS) and added to the calorimetric reaction cell. Stirring was performed at 350 rpm. Each titration experiment was performed with 20 injections of 2.5 µL at 300 s equilibration intervals. The heat of dilution for rhMyD88 protein was determined by titrating it into the PBS. Data were fitted with the NanoAnalyze software package (TA Instruments). The total heat exchanged during each injection of MyD88 to Sch B was fitted to an independent model with variable parameters.

For the pull‐down assays, BeaverBeads Streptavidin and biotinylated‐Sch B were used. Briefly, 100 µL of 1 × 10^−3^ m biotinylated‐Sch B was added to 10 µL streptavidin‐agarose beads and incubated at 25 °C for 2 h. Biotin alone, unbiotinylated Sch B, and untreated beads were used as controls. Lysates prepared from mouse heart tissues were then added to the streptavidin‐agarose beads with Bio‐Sch B. For some studies, lysates prepared from HEK‐293 cells transfected with wildtype MyD88, TIR domain of MyD88, or mutant MyD88 constructs were added. The mixture was incubated at 25 °C for 3 h with gentle rocking. Samples were then spun and washed three times. Eluent was boiled with 5x loading buffer, and the samples were loaded on a 10% polyacrylamide gel for Western Blot analysis. Total lysates were used as an input control.

Bis‐ANS was used to determine rhMyD88 binding to Sch B. Briefly, 5 × 10^−6^ m bis‐ANS and 5 × 10^−9^ m rhMyD88 protein were mixed in PBS and incubated for 15 min to reach stable relative fluorescence units (RFUs), emitted at 430–590 nm under excitation at 385 nm. Sch B was then added at 2.5 × 10^−6^, 5 × 10^−6^, 10 × 10^−6^, or 20 × 10^−6^ m for 5 min and RFU were measured using SpectraMax M5 Multi‐Mode Microplate Reader (Molecular Devices, San Jose, CA). All measurements were done at 25 °C in a 1 cm path‐length quartz cuvette.

### Molecular Docking

The crystal structure of the TIR domain of human MyD88 (PDB code 4EO7) was derived from Protein Data Bank repository. Initial structures of ligand and receptor for docking were prepared by MGLTools 1.5.6 (The Scripps Research Institute, CA, USA).^[^ ^33^ ^]^ Molecular docking was performed by AutoDock Vina 1.0.2.^[^ ^34^ ^]^ The binding conformation of Sch B was sampled on the surface of whole TIR domain of MyD88, and 200 binding poses were obtained. The binding affinity of each docking pose of Sch B was restored by the MM/GBSA method in AmberTools20 package.^[^ ^35^ ^]^ Finally, based on the per‐residue decomposition energy calculations, the key residues for protein‐ligand interaction were identified.

### Experimental Animal Study

All animal care and experimental procedures were approved by the Animal Policy and Welfare Committee in Wenzhou Medical University (Approved number: wydw2019‐0145). 6 weeks old male C57BL/6 mice were obtained from Wenzhou Medical University Animal Centre.

6 weeks old male *db/db* mice (BKS*^db/db^*; stock # T002407) and their male littermate *db/m* (BKS*^db/m^*) mice were purchased from GemPharmatech Co., Ltd. (Nanjing, China). B6/JGpt‐MyD88^flox/flox^ mice (stock # T009598) and B6/JGpt‐H11^Myh6‐Cre^ mice (stock # T004713) were also purchased from GemPharmatech Co., Ltd. (Nanjing, China). All mice were housed at a constant room temperature with a 12/12 h light–dark cycle and fed with a standard rodent diet and water in the Animal Centre of Wenzhou Medical University. The animals were acclimatized to the laboratory for at least 2 weeks before initiating the studies. All animal experiments were performed and analyzed by blinded experimenters. Randomization was used when dividing the groups.

1. *Mice with type 1 diabetes*: Diabetes was induced by intraperitoneal injection of 50 mg kg^−1^ day^−1^ STZ (dissolved in citrate buffer, pH 4.5) for 5 days consecutive. Control group received the same volume of citrate buffer. Control mice received citrate buffer alone. 7 days after STZ injection, mice with a fasting blood glucose concentration greater than 12 mmol L^−1^ were considered diabetic and used in the study. Diabetic mice were randomly divided into the following groups: untreated diabetic mice (T1DM; *n* = 6), diabetic mice treated with 20 mg kg^−1^ Sch B (T1DM+ SchB‐20; *n* = 6), and diabetic mice treated with 40 mg kg^−1^ Sch B (T1DM+ SchB‐40; *n* = 6). Sch B (20 or 40 mg kg^−1^) was administered every other day by oral gavage for 16 weeks. The dosages of Sch B were decided according to previous publications.^[^ ^17^, ^36^ ^]^ Untreated diabetic and nondiabetic control mice received 1% CMC‐Na solution in the same schedule. Blood glucose levels and body weights were recorded regularly.
2. *db/db mice with type 2 diabetes*: 8 weeks old male *db/db* mice (*n* = 18) were used as T2D model, with 8 weeks old male littermates *db/m* mice (*n* = 6) as controls. Mice were maintained at diabetic status for 8 weeks to induced diabetic cardiomyopathy. For Sch B treatment in T2D mice, *db/db* mice were randomized into *db/db* diabetic mice (*db/db*, *n* = 6), diabetic mice treated 20 mg kg^−1^ Sch B (*db/db*+SchB‐20, *n* = 6), diabetic mice treated 40 mg kg^−1^ Sch B (*db/db*+SchB‐40, *n* = 6). Sch B (20 or 40 mg kg^−1^) was administered as oral gavage every 2 days for 8 weeks. The diabetic group and control group received the same volume of 1% CMC‐Na solution in the same schedule. Blood glucose levels and body weights were recorded regularly.
3. *Cardiomyocyte‐specific MyD88 knockout mice*: The cardiomyocyte‐specific MyD88 knockout mice (MyD88^f/f^Myh6^Cre^) were generated using the *Cre‐loxP* method. Mice floxed for MyD88 (MyD88^f/f^) were crossed with mice carrying *Cre‐transgene* under the promoter of Myh6 gene (Myh6‐Cre) that led to the generation of MyD88^f/f^Myh6^Cre^ mice. T1DM in MyD88^f/f^Myh6^Cre^ and MyD88^f/f^ mice (8 weeks old) was also induced by intraperitoneal injection of 50 mg kg^−1^ day^−1^ STZ for 5 consecutive days (MyD88^f/f^Myh6^Cre^+STZ group, *n* = 6; MyD88^f/f^+STZ group, *n* = 6). Mice were maintained at diabetic status for 16 weeks to induced diabetic cardiomyopathy. Control MyD88^f/f^Myh6^Cre^ (*n* = 6) and MyD88^f/f^ mice (*n* = 6) were injected with citrate buffer. Body‐weights and fasting glucose levels were measured in all mice weekly for 16 weeks.

Prior to sacrificing mice, heart function was assessed by Doppler ultrasound. Mice were anesthetized with indrawing 2.5% isoflurane using DRE Compact Mini Rodent Anesthesia Machine once (cat. no. 9280, Avante Animal Health, USA) and then echocardiography was performed by SONOS 5500 ultrasound (Philips Electronics, Amsterdam, Netherland) with a 15 MHz linear array ultrasound transducer. At the end of treatment, mice were sacrificed under 50 mg kg^−1^ sodium pentobarbital by intraperitoneal injection once. The blood and hearts were collected for subsequent analyses.

### Histological Assessments

Paraffin‐embedded, formalin‐fixed sections of heart tissues were used for histological analyses. Sections were stained with Sirius red and Masson's trichrome for assessment of fibrosis. Sections were also stained with H&E for routine histology. For immunohistochemistry, sections were deparaffinized and hydrated. Endogenous peroxidase was blocked with 3% H^2^O^2^ for 30 min. Sections were then incubated with primary antibodies at 1:50 dilution overnight at 4 °C. Secondary antibodies were used at 1:100 for 1 h and immunoreactivity was detected with diaminobenzidine (DAB). All sections were counterstained with hematoxylin.

### NF‐*κ*B Activation and Interferon Responses

H9C2 cells were transfected with NF‐*κ*B and interferon‐stimulated response element (ISRE) reporters (pGL3‐NF‐kB‐EGFP, pGL3‐IRES‐EGFP). Briefly, lentivirus containing the response element of NF‐kB or ISRE was generated by transfecting HEK‐293T cells with pLV‐NF‐kB‐RE‐EGFP or pLV‐ISRE‐EGFP (Inovogen, Beijing, China) plasmids using PEI. Supernatant was collected 48 h later and filtered using a 0.45 mm filter. Then, H9C2 cells were incubated with supernatant and 8 mg mL^−1^ polybrene (Sigma‐Aldrich) for 12 h. Cells were selected with 2 mg mL^−1^ puromycin (Invitrogen).

### Real‐Time Quantitative PCR

Total RNA was isolated from cells and heart tissues using TRIZOL (Invitrogen). Reverse transcription and quantitative PCR were carried out using a two‐step Platinum SYBR Green qPCR SuperMix‐UDG kit (Invitrogen) in an Eppendorf Mastercycler (Eppendorf, Hamburg, Germany). Primers were obtained from Invitrogen (sequences are listed in Table S1, Supporting Information). Target mRNA levels were normalized to *Actb* housekeeping gene.

### Western Blotting and Immunoprecipitation

Proteins were isolated from cells and tissues, and measured by the Bradford assay (Bio‐Rad, Hercules, CA). Proteins were separated by 10% sodium dodecyl sulfate‐polyacrylamide gel electrophoresis and electrotransferred to a polyvinylidene fluoride (PVDF) membrane. Each membrane was blocked for 1.5 h with Tris‐buffered saline containing 0.05% Tween20 and 5% nonfat milk. PVDF membranes were then incubated with specific primary antibodies. Immunoreactive bands were detected by horseradish peroxidase‐conjugated secondary antibodies and visualized using enhanced chemiluminescence (Bio‐Rad). The amounts of the proteins were analyzed using Image J analysis software version 1.38e and normalized to their respective control.

For immunoprecipitation studies, extracts were incubated with antibodies against MD2, TLR4, TLR2, MyD88, or Flag for 4 h and then precipitated with protein G‐Sepharose beads at 4 °C overnight. TLR4, TLR2, MyD88, TRIF, MD2, or HA levels were detected by immunoblotting using specific antibodies.

### Statistical Analysis

All in vitro data represented at least three independent experiments and in vivo data represented at least six independent experiments. All experimental data were expressed as mean ± SEM (standard error of mean). Statistical analyses were performed using GraphPad Pro Prism 8.0 (GraphPad, San Diego, CA). Unpaired two‐tailed Student *t*‐test or one‐way analysis of variance (ANOVA) followed by Bonferroni's multiple comparisons test was employed to analyze the differences between sets of data. *p‐*Value < 0.05 was considered significant.

## Conflict of Interest

The authors declare no conflict of interest.

## Author Contributions

W.L., K.L., and J.H. contributed equally to this work. G.L. and W.L. contributed to the literature search and study design. G.L., Y.W., and W.L. participated in the drafting of the article. W.L., K.L., J.H., J.H., Q.Z., and L.C. carried out the experiments. G.L., Z.A.K., and G.W. revised the manuscript. Y.W. and W.L. contributed to data collection and analysis.

## Supporting information

Supporting Information

## Data Availability

The data that support the findings of this study are available in the Supporting Information of this article.
