# Supplementary material for: Schisandrin B Attenuates Diabetic Cardiomyopathy by Targeting MyD88 and Inhibiting MyD88‐Dependent Inflammation
Source: Adv Sci (Weinh). 2022 Sep 30;9(31):2202590. doi: 10.1002/advs.202202590 (PMC9631063; doi:10.1002/advs.202202590)
Supplement: Supplementary file 1 — Supporting Information [file ADVS-9-2202590-s001.pdf]

Supporting Information

Schisandrin B attenuates diabetic cardiomyopathy via targeting MyD88 and inhibiting MyD88-dependent inflammation

Contents in Supplementary File

Supplementary Table S1, Figures S1-S22, and Legends

Supplementary Table 1: Sequences of primers for real-time qPCR assay used in the study.

Gene	Species	FW	RW
IL-6	Rat	GAGTTGTGCAATGGCAATTC	ACTCCAGAAGACCAGAGCAG
TNF-α	Rat	TACTCCCAGGTTCTCTTCAAGG	GGAGGCTGACTTTCTCCTGGTA
IFN-α	Rat	GACCCAGGAAGACTCCCTACT	GACCCAGGAAGACTCCCTACT
IFN-β	Rat	GACAAAGCACTAGCATTCG	CTCTTCTCCATCTGTGACG
Collagen1	Rat	GACATCCCTGAAGTCAGCTGC	TCCCTTGGGTCCCTCGAC
TGF-β	Rat	AGGAGGAATTTGGCCAGGTG	GCTCACGAGGAGGCTAATCC
β-MyHC	Rat	GAGGAGAGGGCGGACATT	ACTCTTCATTCAGGCCCTTG
α-MyHC	Rat	CGAGTCCCAGGTCAACAAG	AGGCTCTTTCTGCTGGACA
Anp	Rat	GGGCTCCTTCTCCATCACC	CTCCAATCCTGTCAATCCTACC
SKA	Rat	TCCTCCGCCGTTGGCT	AATCTATGTACACGTCAAAAACAGGC
β-actin	Rat	AAGTCCCTCACCCTCCCAAAAG	AAGCAATGCTGTCACCTTCCC
IL-6	Mouse	GAGGATACCACTCCCAACAGACC	AAGTGCATCATCGTTGTTCATACA
TNF-α	Mouse	TGATCCGCGACGTGGAA	ACCGCCTGGAGTTCTGGAA
IFN-γ	Mouse	GCCAAGTTTGAGGTCAACAAC	ATCAGCAGCGACTCCTTTTC
TGF-β	Mouse	TGACGTCACTGGAGTTGTACGG	GGTTCATGTCATGGATGGTGC
Collagen1	Mouse	TGGCCTTGGAGGAAACTTTG	CTTGGAAACCTTGTGGACCAG
Anp	Mouse	AACCTGCTAGACCACCTGGA	TGCTTTTCAAGAGGGCAGAT
β-actin	Mouse	CCGTGAAAAGATGACCCAGA	TACGACCAGAGGCATACAG

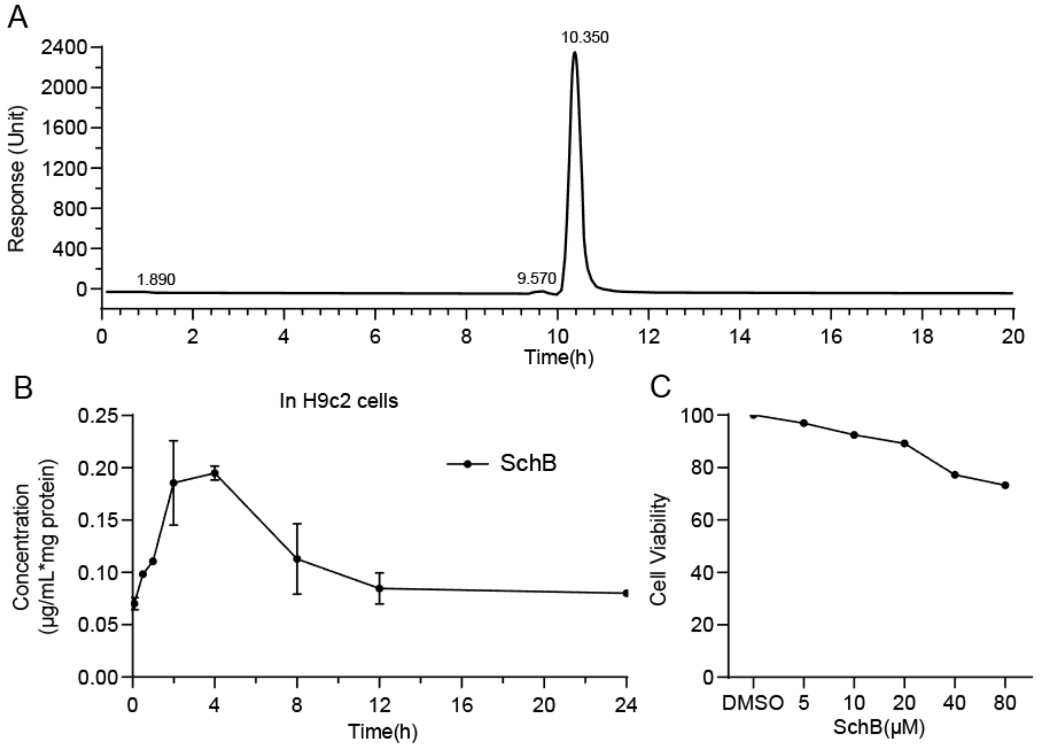

Supplementary Figure S1: Rapid uptake of Sch B in H9c2 cells.

To determine uptake of Sch B, H9c2 cells were treated with 20 μM Sch B dissolved in culture media. At various time points, cells were washed and harvested. Sch B was extracted by adding acetonitrile and concentrations were measured by HPLC using Agilent LC system. The mobile phase consisted of acetonitrile and water (50/50 to 70/30 v/v in 15 min). Chromatographic separation was obtained using Beckman C18 reverse-phase column (5 μm, 4.6 mm x 25 cm) at room temperature and at a flow rate of 1 mL/min. Sch B was monitored at a wavelength of 230 nm. Total proteins were also measured and used to normalize Sch B levels. (**A**) HPLC was used to measure the purity of Sch B (>98.2%) in an Agilent 1260 liquid chromatograph system. (**B**) HPLC analysis showing rapid uptake of Sch B at 20μM from the culture media with peak levels noted at 2-4 hrs. (**C**) Cell viability was assessed following Sch B exposure by MTT assay. Cells were plated in 96-well plates at 5,000 cells per well and treated with Sch B at different concentrations (5, 10, 20, 40, and 80 μM) for 72 hours. Cell viability was calculated using as: cell viability = $A_{treated}$ / $A_{control}$ ×100%. No significant toxicity was noted.

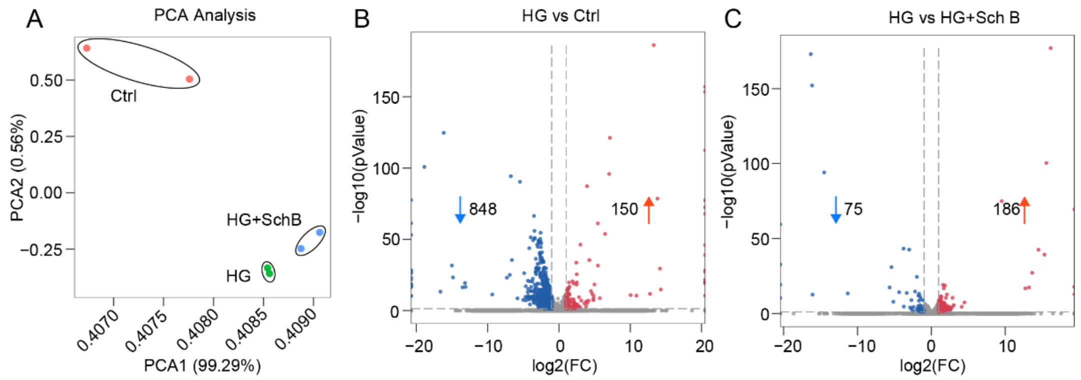

Supplementary Figure S2: H9C2 cells were pretreated with 10 μM Sch B for 1 h before exposure to HG (33 mM) for 8 h. All gene expression levels were detected by RNA sequencing. (A) PCA analysis of all samples. (B, C) Volcano plot analysis of transcriptional changes in Ctrl compared to HG (B), and HG compared to HG+SchB (C). The fold change threshold was set as 2 and P-value ≤ 0.05. Red dots indicate upregulated genes, blue dots indicate downregulated genes, and gray dots indicate non-differentially expressed genes.

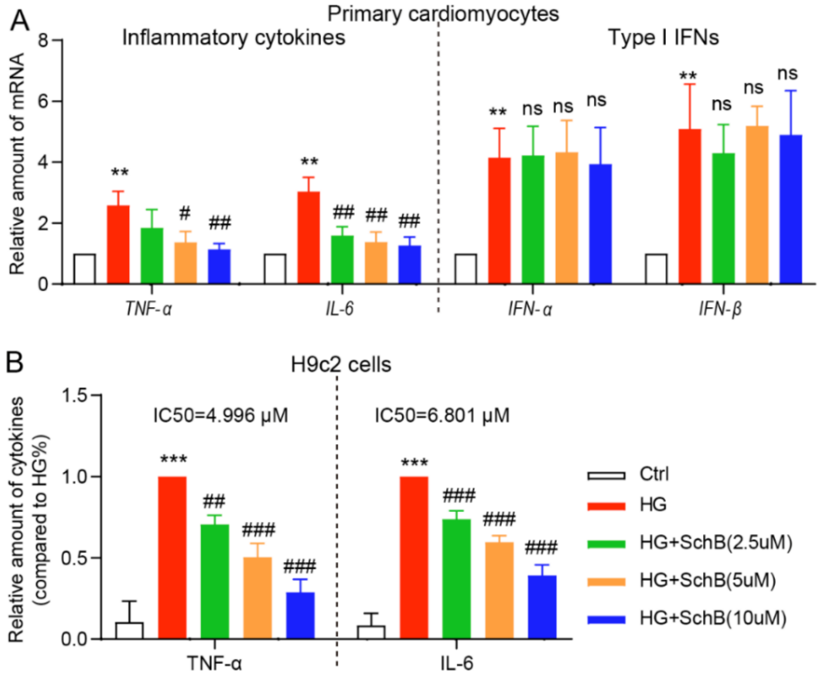

Supplementary Figure S3: Sch B inhibits HG-induced inflammatory cytokines secretion. (A) qPCR verification of classical TLR4 proinflammatory cytokines (TNF-α and IL6) and type 1 interferons (IFN-α and -β) in rat primary cardiomyocytes pretreated with 10 μM Sch B for 1 h before HG exposure for 8h. (B) H9c2 cells were pretreated with Sch B, and were then stimulated with 33 μM HG for 24 h. Medium was collected for ELISA assay to determine relative amount of inflammatory cytokines, including TNF-α and IL-6. Data are shown as Mean ± SEM [n=3; ns = not significant; ## p<0.01; ### p<0.001 compared to HG group; ** p<0.01; *** p<0.001 compared to Ctrl group].

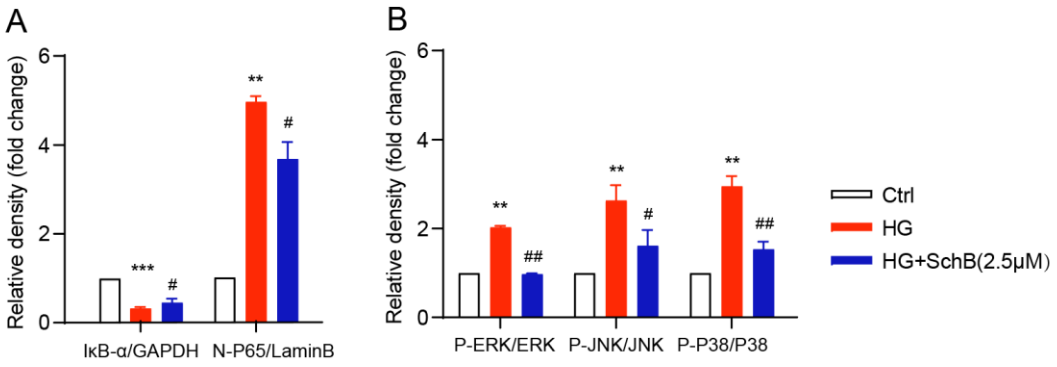

Supplementary Figure S4: (A) Densitometric quantification of the blots in Figure 3B. (B) Densitometric quantification of the blots in Figure 3C. Data are shown as Mean ± SEM; n = 3; ** p<0.01 and ***p<0.001, compared to control group; # p<0.05 and ##p<0.01, compared to HG group.

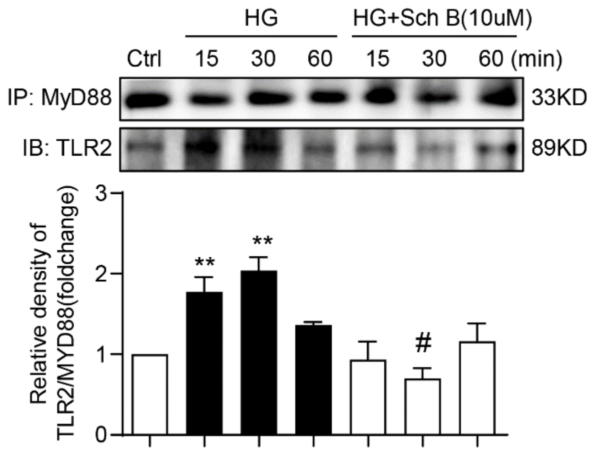

Supplementary Figure S5: Sch B inhibits HG-induced excess interaction between TLR2 and MyD88.

Co-IP analysis of the formation of TLR2/MyD88 complex in H9c2 cells. Cells were pretreated with 10 µM Sch B for 1 h, followed by 33 mM HG stimulation for various time points. TLR2 was immunoprecipitated (IP), and MyD88 were detected by immunoblotting (IB). Lower panel shows the densitometric quantification. Data are shown as Mean ± SEM; n = 3; ** p<0.01 and ***p<0.001, compared to control group; # p<0.05, compared to HG group (30min).

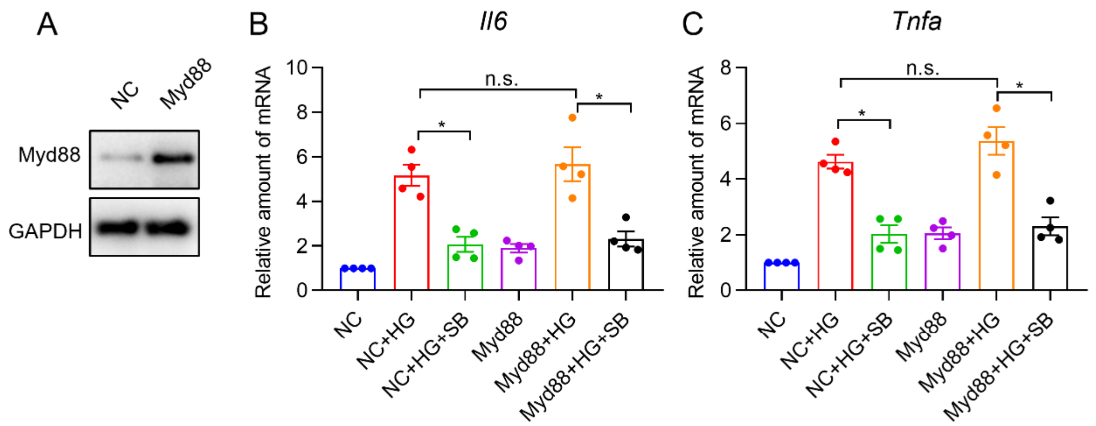

Supplementary Figure S6: Sch B reversed HG-induced *Il6* and *Tnfa* gene transcription in H9c2 cells with MyD88 overexpression. (A) Western blot analysis of MyD88 overexpression in H9C2 cells. Cells were transfected with empty vector (NC) or HA-tagged MyD88 plasmid (MyD88). GAPDH was used as loading control. (B-C) qPCR analysis of inflammatory cytokines (*Il-6* and *Tnfa*) in H9C2 cells transfected with empty vector or HA-tagged MyD88 plasmid. Transfected cells were treated with 10 μM Sch B for 1 h before exposure to HG for 8 h. [Values represent Mean ± SEM; n = 4; ns = not significant; *p<0.05, Statistical analysis of differences between groups was employed to 2-tailed Student's t test].

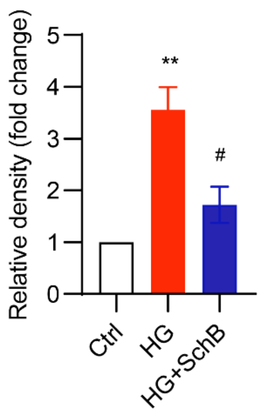

Supplementary Figure S7: Densitometric quantification of the blots in Figure 4C. Data are shown as Mean ± SEM; n = 3; ** p<0.01, compared to control group and # p<0.05, compared to HG group by one-way ANOVA followed by Bonferroni's multiple comparisons test.

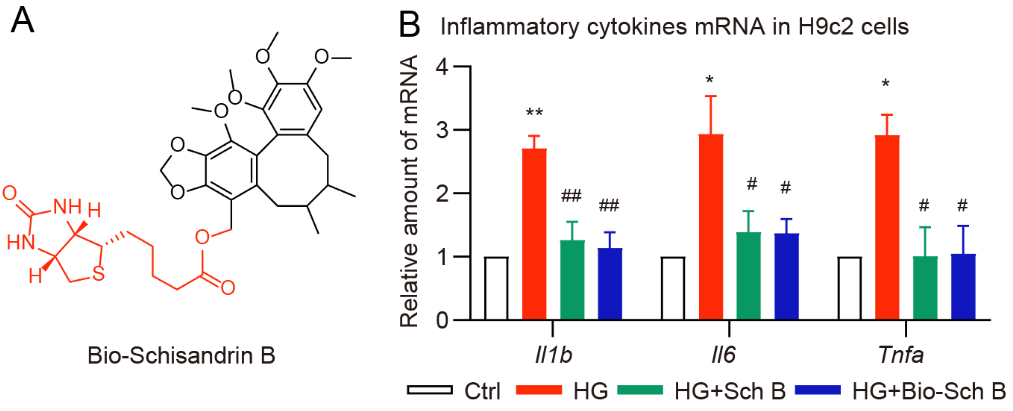

Supplementary Figure S8: Biotinylated-Sch B retains anti-inflammation activity as Sch B.
(A) Chemical structure of Biotinylated-Sch B (Bio-Sch B). (B) Real time qPCR analysis. H9c2 cells were pretreated with 10 μM Sch B or 10 μM Bio-Sch B for 1 h, and then stimulated with 33 μM HG for 8 h, and mRNA levels of *Il1b*, *Il6* and *Tnfa* were detected. Data are shown as Mean ± SEM; n = 3; *p<0.05 and ** p<0.01, compared to control group; # p<0.05 and ##p<0.01, compared to HG group by one-way ANOVA followed by Bonferroni's multiple comparisons test.

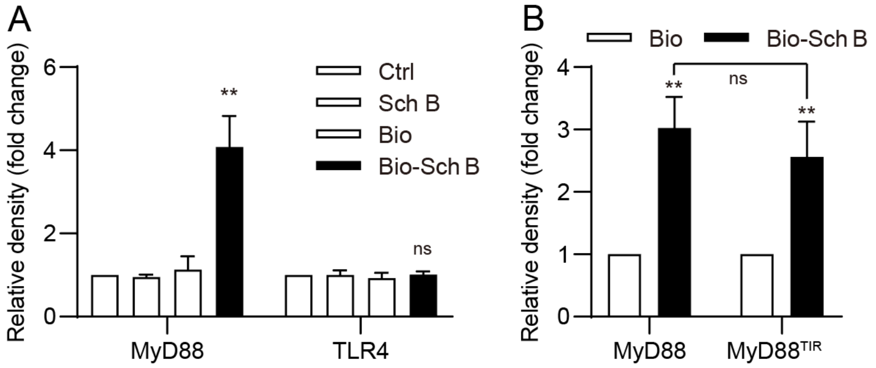

Supplementary Figure S9: Sch B directly binds to TIR domain of MyD88.
(A) Densitometric quantification of the blots in Figure 4D. [**, compared to control group, p<0.01; ns = not significant, compared to control group]. (B) Densitometric quantification of the blots in Figure 4E. Data are shown as Mean ± SEM; n = 3; ** p<0.01 and ns= not significant, compared to control (and Bio) group.

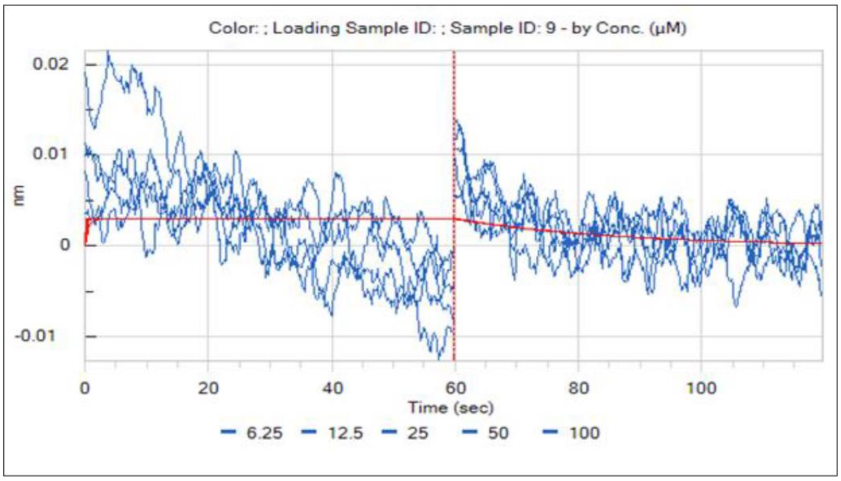

Supplementary Figure S10: Sch B does not bind to rhTLR4.

SPR analysis was performed to determine whether SB binds to recombinant human TLR4 protein (rhTLR4). SB was added at different concentrations and no binding signal was detected.

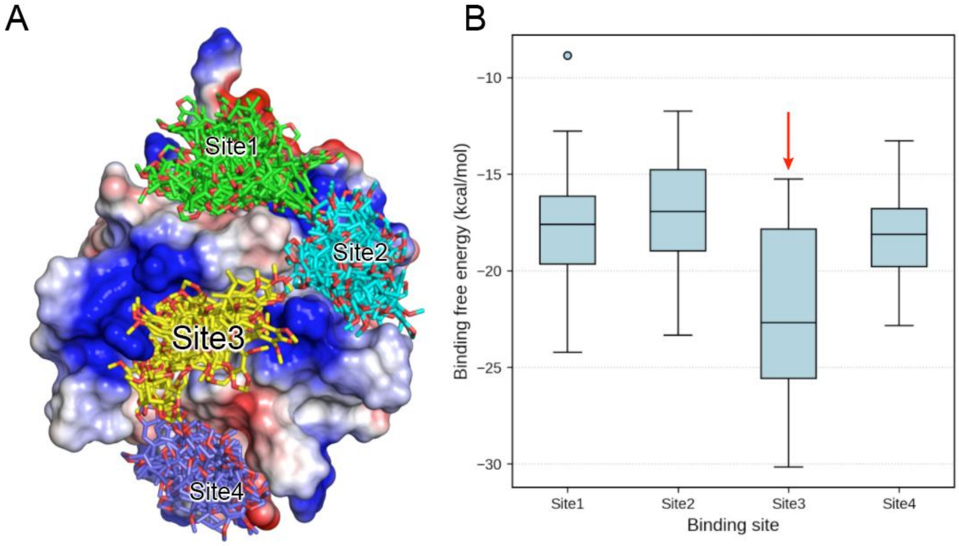

Supplementary Figure S11: Molecular docking shows the possible binding site of MyD88 with Sch B. (A) Four possible binding sites of schisandrin B on the TIR domain of MyD88. (B) The boxplot of docking scores of schisandrin B at four different binding sites, respectively.

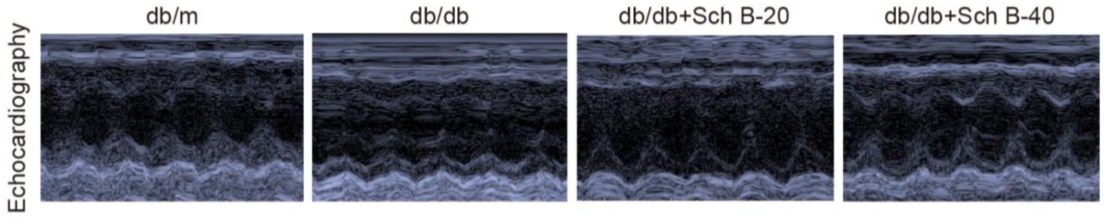

Supplementary Figure S12: Representative left ventricular M-mode echocardiographic tracings at 16th week. [db/m, non-diabetic controls, n=6; db/db, untreated diabetic mice n=6; db/db+SB-20, diabetic mice treated 20 mg/kg SB, n=6; db/db+SB-40, diabetic mice treated 40 mg/kg SB, n=6.

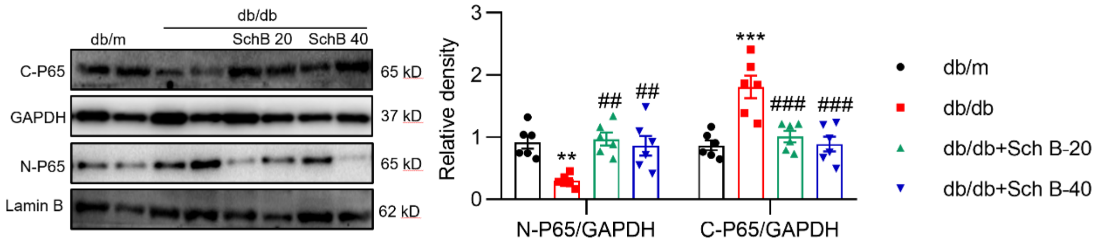

Supplementary Figure S13: Western blot analysis of nuclear translocation of NF-κB p65 in hearts of T2DM mice. p65 in nuclear fractions (N-P65) and p65 in cytoplasmic fractions (C-P65) were prepared and detected. GAPDH was used as loading control for cytoplasmic proteins and lamin B for nuclear proteins. Densitometric quantification shown on right. Data are shown as Mean ± SEM; n = 6; ** p<0.01, *** p<0.001, compared to db/m group and ## p<0.01, ### p<0.001, compared to db/db group by one-way ANOVA followed by Bonferroni's multiple comparisons test.

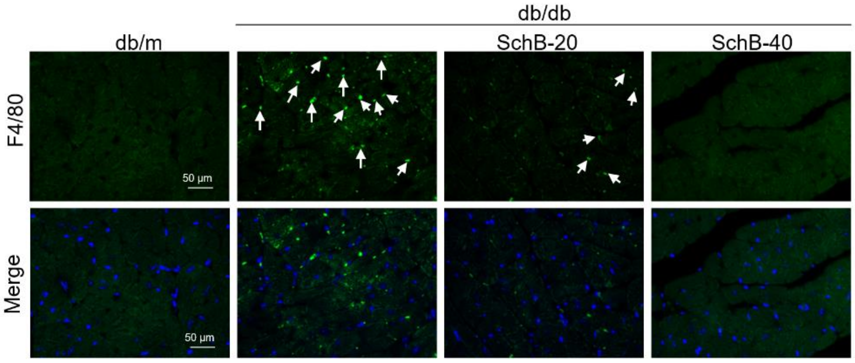

Supplementary Figure S14: Representative F4/80 immunostaining images of T2DM mouse heart tissues. [scale bar = 50 μm].

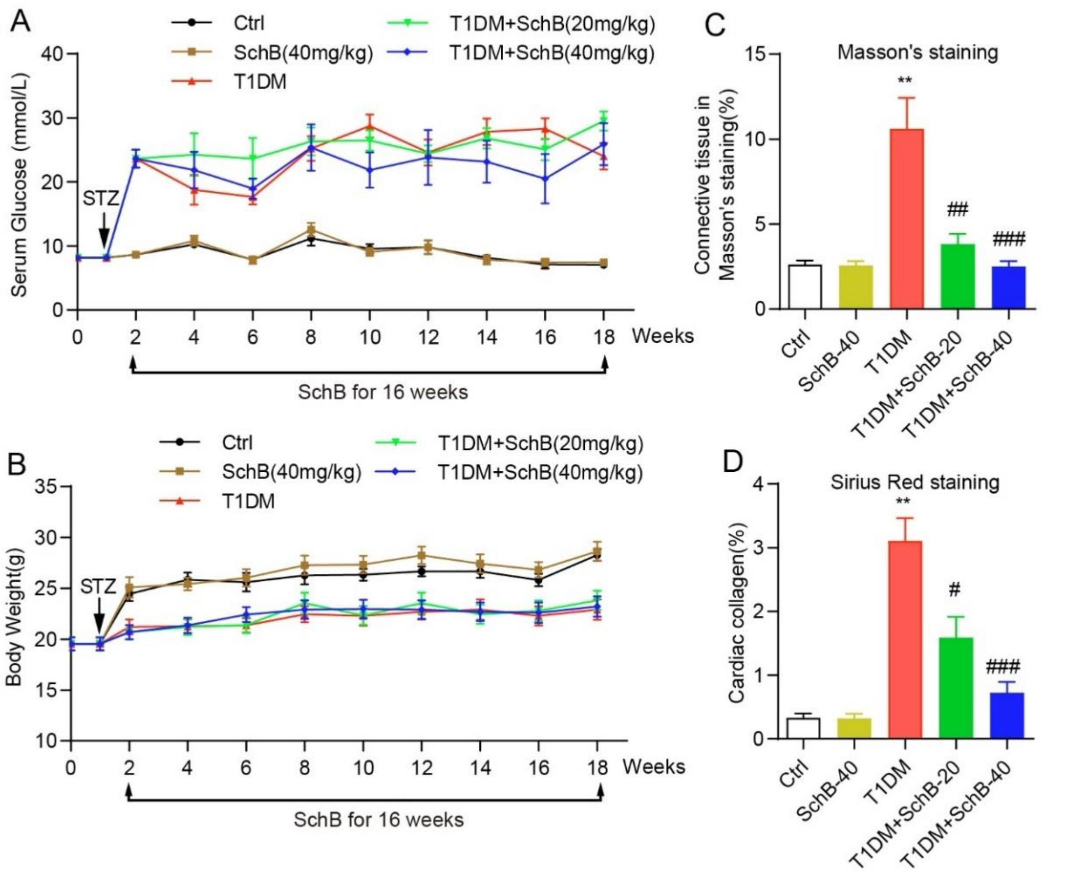

Supplementary Figure S15: Sch B improves cardiac injury in STZ-induced T1DM.

Mice were made diabetic by a single intraperitoneal of streptozotocin (STZ) at the first week. Mice were given Sch B or vehicle solution a week after the STZ injection. After 18 weeks, mice were sacrificed and samples were collected for further assay. (A) Blood glucose of mice in indicated time points. (B) Body weight of mice in indicated time points. (C) Quantifications of Masson's staining in Figure 7A. (D) Quantifications of Sirius Red staining in Figure 7B. Data are shown as Mean ± SEM; n = 6 per group; ** $p<0.01$, compared to control group; # $p<0.05$, ##$p<0.01$, and ###$p<0.001$, compared to T1DM group.

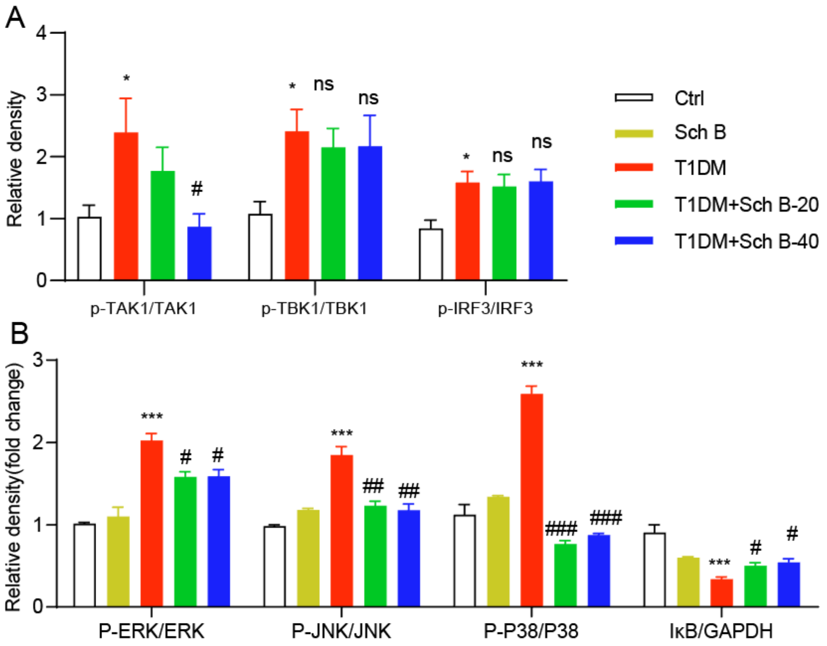

Supplementary Figure S16: Sch B inhibits the activation of MyD88 downstream pathway in the heart tissues of T1DM.

(A) Densitometric quantifications of blots in Figure 7F. (B) Densitometric quantifications of blots in Figure 7G. Data are shown as Mean ± SEM; n = 6 per group; *p<0.05, ***p<0.001, ns = not significant, compared to control group; # p<0.05, ##p<0.01, and ###p<0.001, compared to T1DM group.

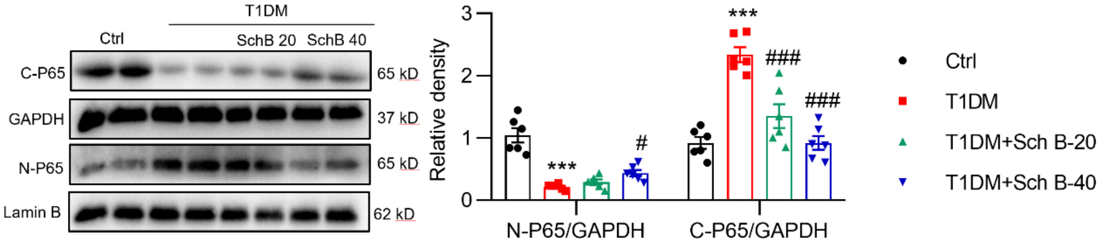

Supplementary Figure S17: Western blot analysis of nuclear translocation of NF-κB p65 in hearts of T1DM mice. p65 in nuclear fractions (N-P65) and p65 in cytoplasmic fractions (C-P65) were prepared and detected. GAPDH was used as loading control for cytoplasmic proteins and lamin B for nuclear proteins. Densitometric quantification shown on right. Data are shown as Mean ± SEM; n = 6; *** p<0.001, compared to ctrl group and # p<0.05, ### p<0.001 compared to T1DM group by one-way ANOVA followed by Bonferroni’s multiple comparisons test.

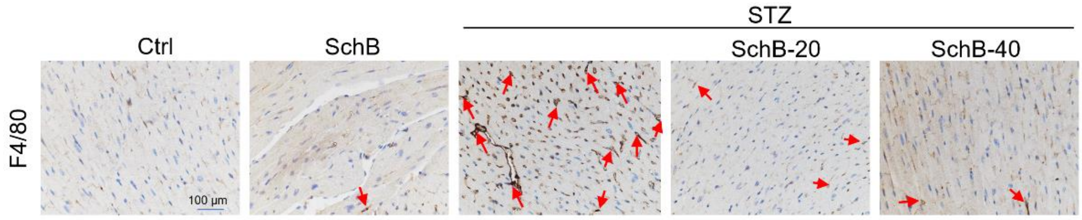

Supplementary Figure S18: Representative F4/80 immunostaining images of T1DM mouse heart tissues. [scale bar = 100 µm].

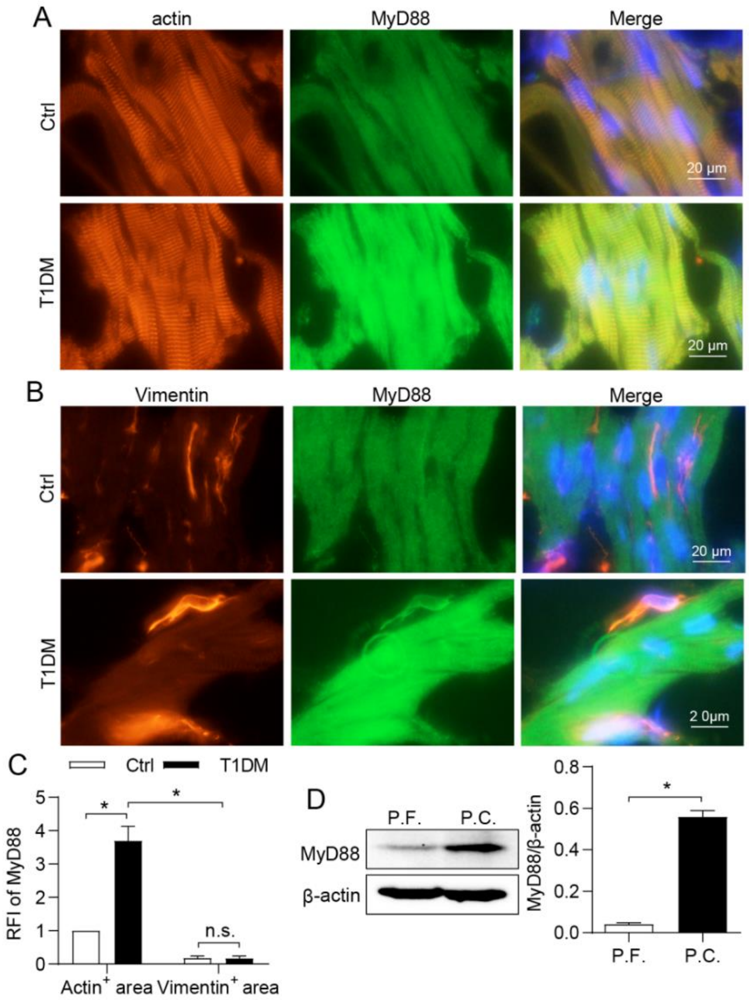

Supplementary Figure S19: MyD88 expression was up-regulated in cardiomyocytes of diabetic mice. (**A**) Representative images of the immunofluorescent double-staining for MyD88 (green) and cardiomyocyte-marker actin (orange). **(B)** Representative images of the immunofluorescent double-staining for MyD88 (green) and fibroblast-marker vimentin (orange). **(C)** Quantification for panel C and D, shown by the relative fluorescence intensity (RFI) fold compared to the MyD88-positive area. **(D)** Western blot analysis for MyD88 protein in primary myocardial fibroblasts (P.F.) and primary cardiomyocytes (P.C.) of neonatal rats. β-Actin was used as loading control. Densitometric quantification was shown in right. Data were mean ± SEM. Scale bar = 20 µm; n = 5 per group; *P<0.05; n.s. = not significant.

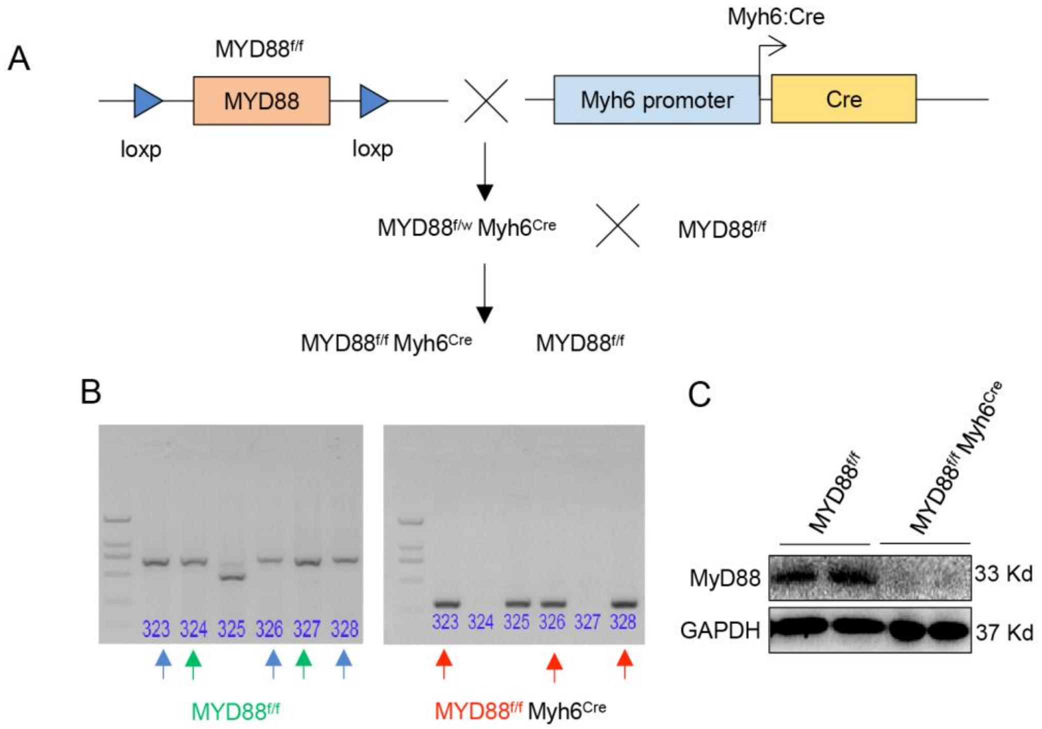

Supplementary Figure S20: Generation of cardiomyocyte-specific MyD88 knockout mouse. (A) Diagram depicting the generation of cardiomyocyte-specific MyD88 knockout mice ($MyD88^{f/f}$-$Myh6^{Cre}$) using the *Cre-loxP* method. Mice floxed for MyD88 ($MyD88^{f/f}$) were crossed with mice carrying *Cre-transgene* under the promoter of Myh6 gene (Myh6-Cre) that led to the generation of $MyD88^{f/f}$-$Myh6^{Cre}$ mice. $MyD88^{f/f}$-$Myh6^{Cre}$ mice were further crossed with $MyD88^{f/f}$ mice to generate $MyD88^{f/f}$-$Myh6^{Cre}$ mice. (B) Genotyping was performed using genomic DNA isolated from 3-weeks old mice and *loxP* or *Cre* sequence-specific primers by PCR. The PCR products were separated using agarose gel electrophoresis to identify PCR products of 484 bp ($MYD88^{w/w}$), 689 bp ($MYD88^{f/f}$), 484/689 bp ($MYD88^{f/w}$) and 300 bp ($Myh6^{Cre}$). (C) Immunoblot for MyD88 performed on whole heart lysates obtained from 6-months old $MYD88^{f/f}$ and $MyD88^{f/f}$-$Myh6^{Cre}$ mice demonstrate successful knockdown of MyD88 in the heart of $MyD88^{f/f}$-$Myh6^{Cre}$ mice. GAPDH was used as a loading control for immunoblotting.

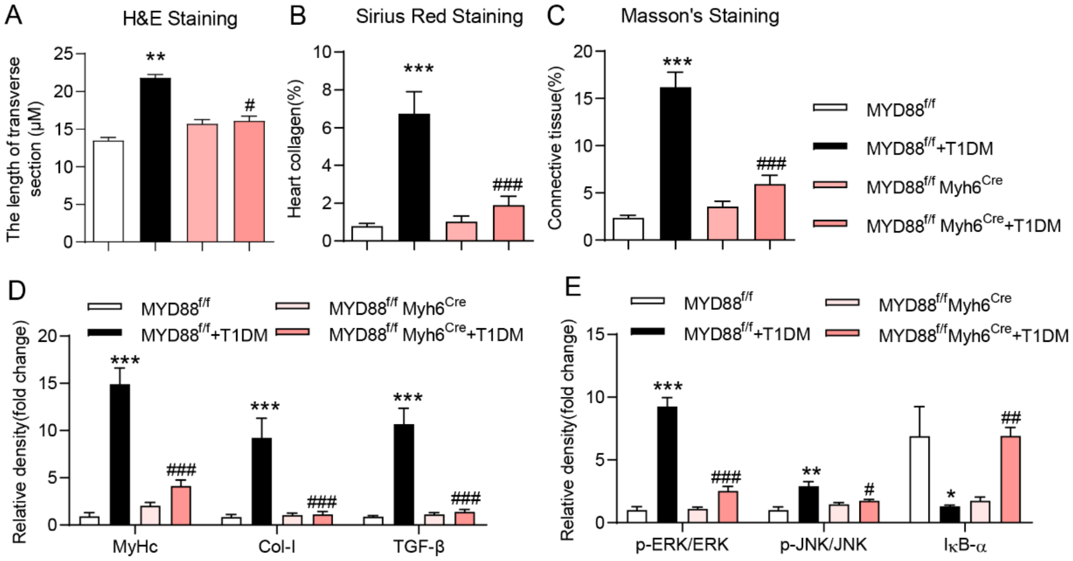

Supplementary Figure S21: (A) The bar graph shows the quantification of myocardial cell size (the representative staining images are presented in Figure 8F). (B) Quantification of Sirius red staining in Figure 8G. (C) Quantification of Masson's Trichrome staining in Figure 8G. (D) Densitometric quantification of blots in Figure 8H. (E) Densitometric quantification of blots in Figure 8J. Data are shown as Mean ± SEM; n = 6 per group; * $p<0.05$, ** $p<0.01$, *** $p<0.001$, compared to MyD88$^{f/f}$ group; # $p<0.05$, ## $p<0.01$, ### $p<0.001$, compared to MyD88$^{f/f}$+T1DM group by one-way ANOVA followed by Bonferroni's multiple comparisons test.

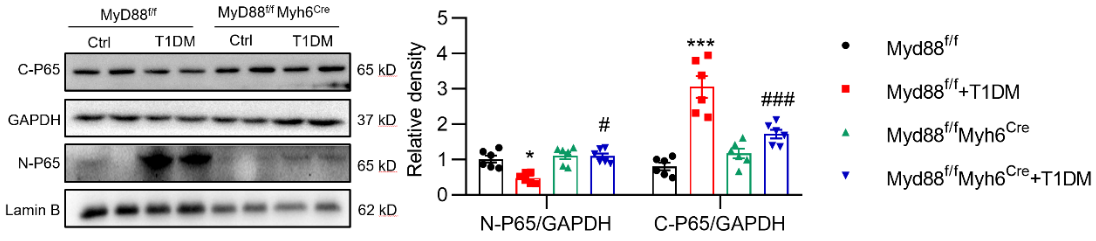

Supplementary Figure S22: Western blot analysis of nuclear translocation of NF-κB p65 in hearts of cardiomyocyte-specific MyD88 knockout mouse. p65 in nuclear fractions (N-P65) and p65 in cytoplasmic fractions (C-P65) were prepared and detected. GAPDH was used as loading control for cytoplasmic proteins and lamin B for nuclear proteins. Densitometric quantification shown on right. Data are shown as Mean ± SEM; n = 6; * $p<0.05$, *** $p<0.001$, compared to Myd88$^{f/f}$ group and # $p<0.05$, ### $p<0.001$ compared to Myd88$^{f/f}$+T1DM group by one-way ANOVA followed by Bonferroni's multiple comparisons test.
